# Structure of the actively translating plant 80S ribosome at 2.2 Å resolution

**DOI:** 10.1038/s41477-023-01407-y

**Published:** 2023-05-08

**Authors:** Julia Smirnova, Justus Loerke, Gunnar Kleinau, Andrea Schmidt, Jörg Bürger, Etienne H. Meyer, Thorsten Mielke, Patrick Scheerer, Ralph Bock, Christian M. T. Spahn, Reimo Zoschke

**Affiliations:** 1 Institute of Medical Physics and Biophysics, Charité—Universitätsmedizin Berlin, corporate member of Freie Universität Berlin and Humboldt-Universität zu Berlin, Berlin, Germany; 2 Institute of Medical Physics and Biophysics, Group Protein X-ray Crystallography and Signal Transduction, Charité—Universitätsmedizin Berlin, corporate member of Freie Universität Berlin and Humboldt-Universität zu Berlin, Berlin, Germany; 3 Microscopy and Cryo-Electron Microscopy Service Group, Max Planck Institute for Molecular Genetics, Berlin, Germany; 4 Department III, Max Planck Institute of Molecular Plant Physiology, Potsdam-Golm, Germany; 5 Present Address: Institut für Pflanzenphysiologie, Martin-Luther-Universität Halle-Wittenberg, Halle (Saale), Germany

**Keywords:** Plant molecular biology, Cryoelectron microscopy

## Abstract

In plant cells, translation occurs in three compartments: the cytosol, the plastids and the mitochondria. While the structures of the (prokaryotic-type) ribosomes in plastids and mitochondria are well characterized, high-resolution structures of the eukaryotic 80S ribosomes in the cytosol have been lacking. Here the structure of translating tobacco (*Nicotiana tabacum*) 80S ribosomes was solved by cryo-electron microscopy with a global resolution of 2.2 Å. The ribosome structure includes two tRNAs, decoded mRNA and the nascent peptide chain, thus providing insights into the molecular underpinnings of the cytosolic translation process in plants. The map displays conserved and plant-specific rRNA modifications and the positions of numerous ionic cofactors, and it uncovers the role of monovalent ions in the decoding centre. The model of the plant 80S ribosome enables broad phylogenetic comparisons that reveal commonalities and differences in the ribosomes of plants and those of other eukaryotes, thus putting our knowledge about eukaryotic translation on a firmer footing.

## Abstract (web summary)

A 2.2 Å cryo-electron microscopy structure of an actively translating 80S ribosome from tobacco reveals bound tRNA and mRNA, the nascent peptide and numerous cofactors, providing a basis for understanding the molecular mechanism of protein synthesis in plants.

## Main

The regulation of gene expression is fundamental to all organisms in responding properly to their dynamically changing environment. Plants are sessile and have evolved multifaceted regulatory mechanisms for rapid acclimation to the alterations of diverse environmental factors that they cannot escape from. Translation represents a unique opportunity for rapid regulation of gene expression in eukaryotes^1^. In plants, extensive regulation at the level of mRNA translation into protein enables the fast adjustment of gene expression to environmental cues^2–4^.

The macromolecular machines that execute protein biosynthesis, the ribosomes, consist of rRNA and ribosomal proteins (RPs). To maintain the three-dimensional rRNA structure required to perform the peptidyl transferase reaction, ribosomes require small-molecule cofactors such as ions and polyamines^5,6^. Moreover, chemical modifications of the rRNA are ubiquitously present in eukaryotic rRNAs and are fundamental to ribosome structure and function^7,8^.

In plants, protein synthesis is realized by eukaryotic 80S ribosomes in the cytosol and 70S-type ribosomes of prokaryotic origin in chloroplasts and mitochondria^9^. High-resolution structures for chloroplast and plant mitochondrial 70S-like ribosomes have been reported recently^10,11^. Likewise, numerous structures of 80S ribosomes from ‘non-green’ species are available^12–17^. However, for the whole plant kingdom, the outdated structure of the wheat (*Triticum aestivum*) 80S ribosome that was resolved to 5.5 Å has been the only available standard for many years^18^. Only very recently, an independent 80S ribosome structure from a tomato plant (*Solanum lycopersicum*) became available^19^. Hence, the lack of high-resolution structures of a plant 80S ribosome and functional ribosomal complexes represents an obvious critical gap in our knowledge of translation.

Even more importantly, the ribosome is a highly dynamic machine that undergoes numerous intra- and intermolecular rearrangements during translation. These include intersubunit rotation, the movement of mRNA and tRNA molecules through the ribosome, and the association and dissociation of various translation factors and cofactors such as ions and small molecules^20–22^. All of these numerous distinct ribosomal structures have not been determined in plants. This knowledge gap has also hindered a profound understanding of plant-specific mechanisms of translational regulation^23–25^, which have important implications for plant growth and development. Obtaining structural information on key intermediates of the plant 80S ribosome during translation is thus of utmost importance.

To elucidate the structural basis of cytosolic translation in plants, we report the cryo-electron microscopy (cryo-EM) structure of actively translating cytosolic 80S ribosomes in a rotated conformation (rotated-2 state) from the model plant tobacco (*Nicotiana tabacum*) at a global resolution of 2.2 Å. This resolution allowed us not only to build the correct atomic coordinates for rRNA and RPs in the 80S ribosome but also to describe ribosome solvation and metal ion positions, as well as chemical modifications of rRNA residues. Our structure also provides molecular details of interactions between bound ligands (tRNAs, mRNA and the nascent peptide chain) and the ribosome during the elongation phase of translation. Moreover, the structure presented here uncovers plant-specific features of 80S ribosomes and allows us to assess the phylogenetic conservation of eukaryote-specific elements in ribosome structure.

## Results

### Cryo-EM reconstruction of a translating plant 80S ribosome

To elucidate features of the plant eukaryotic translation machinery, the structure of the tobacco 80S ribosome was solved in a native functional state. To this end, polysomes (that is, actively translating ribosomes in association with mRNAs) were isolated from freshly harvested tobacco leaves and used for cryo-EM (Supplementary Fig. 1).

Multiparticle refinement^22,26^ identified two main populations of ribosomes in our leaf samples, 80S and 70S-like ribosomes (Supplementary Fig. 2), which were assigned to the cytosolic and chloroplast ribosome pools, respectively. Cytosolic and chloroplast ribosomes account for ~70% and 30% of all ribosomal particles in our sample, respectively, which is consistent with previous biochemical examinations in plants^9^.

Elongating 80S ribosomes can adopt a variety of functional states and typically consist of a mixture of 80S ribosomes with classical or rotated intersubunit arrangements as well as tRNAs in different positions and states^22^. Further multiparticle refinement at intermediate resolution resulted in several 80S species with bound ligands such as tRNA, mRNA and the nascent chain (NC), which represent different intermediates of the elongation cycle similar to those obtained for mammalian cells^22,26^ (Supplementary Figs. 2 and 3). These results confirm that our sample consists of actively translating ribosomes.

**Fig. 1:**
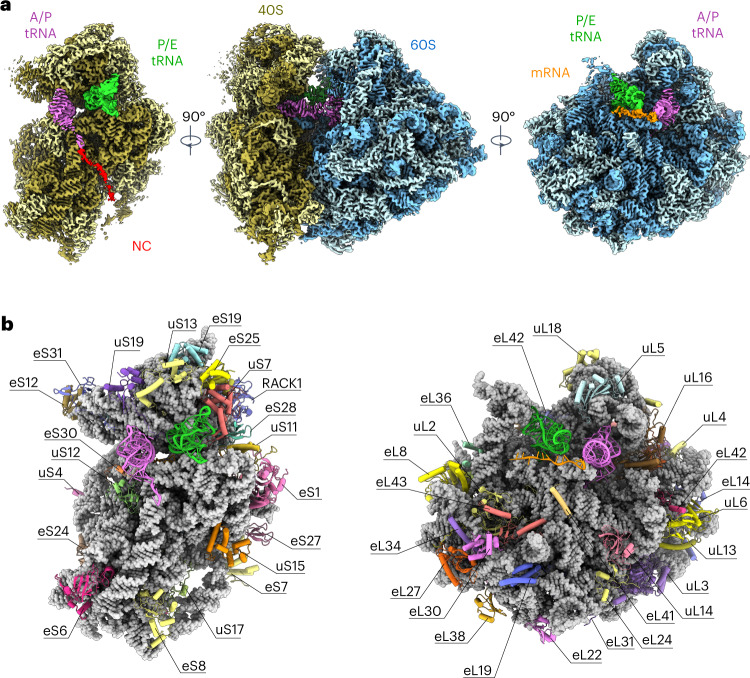
High-resolution structure and atomic model of the tobacco 80S ribosome in the rotated state. **a**, Cryo-EM density map of the actively translating cytosolic ribosome from tobacco in the rotated-2 state with bound tRNAs at 2.2 Å resolution. The 60S rRNA is shown in dark blue, 60S RPs are light blue, the 40S rRNA is dark yellow, 40S RPs are light yellow, A/P tRNA is pink, P/E tRNA is green, mRNA is orange and the NC is red. The reconstruction is shown from the intersubunit side with the 60S subunit computationally removed (left), from the A-site region (middle) and from the intersubunit side with the 40S subunit computationally removed (right). The NC is shown on the 40S ribosomal subunit for visual clarity only, as the path of the NC through the exit tunnel is obstructed by the surrounding density of the 60S subunit. **b**, The newly built atomic models for the ribosomal 40S (left) and 60S (right) subunits with bound ligands are shown from the intersubunit side. The individually coloured RPs are shown as ribbons, the rRNAs are shown in grey as spheres, and the tRNAs and mRNA are shown as ribbons with nucleotides in ladder representation. A/P tRNA is shown in pink, P/E tRNA is green and mRNA is orange.

To obtain a dataset suitable for high-resolution structure determination, we then extracted the largest subpopulation, representing the pre-translocational rotated-2 state^16,20,22^ (Supplementary Figs. 2 and 3). The global resolution of the whole complex reached 2.2 Å. Further local refinement of the 60S and 40S subunits improved the local resolution and overall map quality, especially for the 40S subunit, which is known to be particularly dynamic^20^ (Supplementary Fig. 4). The resulting small subunit (SSU) and large subunit (LSU) structures and the cryo-EM map of the entire 80S complex with bound ligands were subsequently used to model the actively translating 80S ribosome in the pre-translocational rotated-2 state, including tRNA-binding pockets and intersubunit bridges (Fig. 1a and Supplementary Table 1). Despite the expected codon heterogeneity in our ex vivo 80S ribosome, the translated mRNA chain was well resolved for at least 12 nucleotides. Likewise, the densities for both bound tRNA molecules were of sufficient quality to build their atomic models, to exemplify the structure of bound tRNAs despite the mixture of tRNA species expected in the ex vivo specimen.

### Modelling the structure of the plant 80S ribosome

The Sol Genomics Network (SGN) database^27^ was used to obtain the complete rRNA sequences of the 80S tobacco ribosome (Supplementary Table 2). Our cryo-EM map confirmed the SGN-annotated 3′ and 5′ ends of 25S, 18S, 5.8S and 5S tobacco rRNAs, which are largely conserved compared with those of *Arabidopsis* (Supplementary Fig. 5).

In plants, RPs are usually encoded by multiple paralogous genes (two to six in *Arabidopsis*) that form a small RP gene family^28^. The number of paralogous genes within each RP family is further increased in tobacco due to its allotetraploid genome^29^. To obtain detailed information about the extent of proteomic heterogeneity within the tobacco 80S ribosome pool, we performed mass spectrometry (MS) of the polysome sample. Overall, the MS data returned an almost complete set of 80 RP families out of 81 RP families that have been described in the 80S ribosome of the vascular plant *Arabidopsis*^30^. Only protein eL41 from the LSU was not detected by MS, probably due to its small size (25 amino acids). However, a clear density for this RP unambiguously confirmed its presence in the tobacco 80S ribosome (Fig. 1). Within the tobacco RP pool, we revealed extensive heterogeneity with two or more paralogous proteins identified for the vast majority of the detected 80 RP families (Supplementary Tables 3 and 4). The MS data showed that paralogous RPs have variable lengths and/or amino acid compositions, but these differences are very minor. Although the resolution of our cryo-EM map would, in principle, enable the discrimination of RP isoforms, this has not been pursued due to the large number of combinations. Consequently, the final structure reflects a mixture of highly similar but not identical RP isoforms, which is due to averaging hundreds of thousands of single-particle images for the modelled structure. To model the consensus plant 80S ribosome, one protein isoform from the list of paralogues was chosen (virtually randomly selected with preference for long protein isoforms, to prevent the exclusion of amino acid residues from the density maps; Supplementary Tables 3 and 4). In total, 91% of the rRNA residues and all 33 RPs within the SSU as well as 95% of the rRNA residues and 43 (of 48) RPs within the LSU are present in our model, while a few surface-exposed elements that exhibit flexible structures have been excluded (Fig. 1b and Supplementary Tables 1, 3 and 4).

In addition, the solvation of the tobacco 80S ribosome has been modelled, including water molecules, polyamines, magnesium (Mg^2+^) and other monovalent ions (Supplementary Table 1 and Supplementary Figs. 6–8). The presented maps of the ribosomal 40S and 60S subunits provide multiple high-resolution details, also allowing the elucidation of the role of chemical modifications of rRNAs in the plant 80S ribosome (Supplementary Fig. 9).

### Transfer RNA interactions in the plant 80S ribosome

**Fig. 2:**
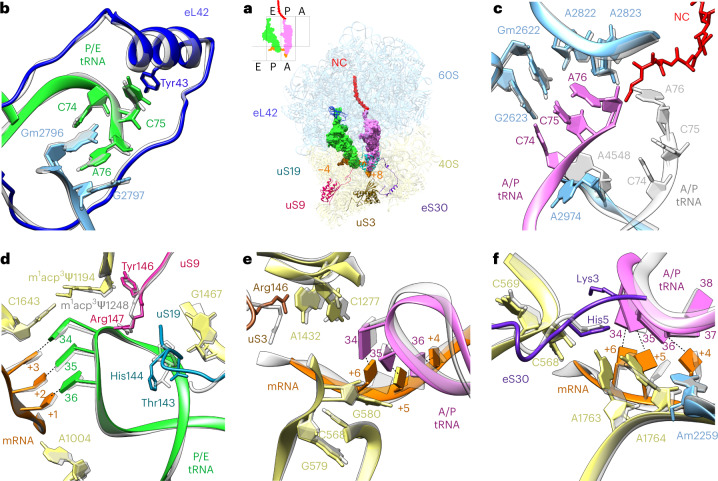
Comparison of tobacco and human ribosomal binding pockets with bound tRNAs. **a**, Overview of the tobacco ribosome in the rotated state with two tRNAs. **b**–**f**, Key interactions of tRNAs with their binding pockets in the large (**b**,**c**) and small (**d**–**f**) subunits. The small schematic shows the positions of the tRNAs in the rotated-2 state. RPs involved in tRNA stabilization are annotated in the model. The large (60S, blue) and small (40S, yellow) subunits are shown along with eL42 (dark blue), uS19 (cyan), uS9 (magenta), uS3 (brown), eS30 (purple), A/P-site tRNA (pink), P/E-site tRNA (green), mRNA (positions −4 to +8, orange) and the NC (red). The atomic model of tobacco is shown in colour; the human model (Protein Data Bank (PDB): 6y57) is underlaid in grey. The details of the molecular interactions shown here are summarized in Supplementary Table 5.

The polysome purification protocol we used delivers actively translating ribosomes; accordingly, the reconstruction of the largest 80S subpopulation showed bound tRNAs in A/P and P/E hybrid positions as well as mRNA and an NC (Fig. 1a). This state corresponds to a rotated-2 pre-translocational state (Supplementary Fig. 3), which enables the examination of key interactions between the ribosome and bound tRNAs and mRNAs (Fig. 2a and Supplementary Table 5).

For both A/P and P/E tRNAs, a codon–anticodon interaction with the mRNA was observed (Fig. 2d–f). In the decoding centre, a tight network of several ribosomal components facilitates the correct matching of mRNA codon and aminoacyl-tRNA anticodon during translation (Fig. 2e,f and Supplementary Table 5). The first nucleotide of the anticodon (position 34 of the aminoacyl-tRNA) is stabilized by the tight stacking of nucleotides C1277 and A1432 of the 18S rRNA and Arg146 of the uS3 protein (Fig. 2e). The second nucleotide of the aminoacyl-tRNA anticodon (position 35) is stabilized by the stacking of nucleotides of the 18S rRNA 530 loop (G579, G580 and C569 sandwiched between the two). The third nucleotide (position 36) interacts with A1763 and A1764 of the 18S rRNA, both in flipped-out position, corresponding to an accommodated state^31^ (Fig. 2f). The interaction between the tRNA anticodon stem–loop (36–37) and the 18S rRNA 530 loop (C568 and C569) is additionally promoted by the amino terminus of eS30 via Lys3 and His5, as has been proposed for the human pre-translocation complex^20^ (Fig. 2f).

The codon–anticodon interaction between mRNA and tRNA at the P site is supported by 18S rRNA elements as well as by proteins (Fig. 2d and Supplementary Table 5). Similar to a human ribosome^22^, the carboxy-terminal residue Arg147 of protein uS9 directly interacts with the anticodon—namely, with phosphates of nucleotides 33 and 35. In addition, the neighbouring Tyr146 of protein uS9 interacts with 18S rRNA U1585, C1586 and A1163, thus playing an important role in stabilizing the codon–anticodon interaction. Moreover, in the tobacco ribosome structure, the C terminus of uS19 is nicely resolved and reveals interactions with the P/E tRNA anticodon arm, apparently supporting accommodation and translocation during the elongation cycle (Fig. 2d). Recently, it was shown in human 80S that the C-terminal tail of uS19 dynamically interacts with A- and P-site tRNAs, suggesting its role in stabilizing the interactions of tRNAs with the SSU during translation elongation^16,32^.

In the LSU, the NC was traced and modelled as continuous density extending from the P-site tRNA to the end of the exit tunnel, similar to a human 80S ribosome^22^ (Fig. 2c). Moreover, in our structure, the 3′-CCA end of the A/P tRNA is nicely resolved, enabling unambiguous visualization of its interaction with the peptidyl transferase centre. The stacking of residues C74, C75 and A76 and the formation of Watson–Crick base pairs of C74 and C75 with residues of the 25S rRNA (Gm2622 and G2623) are observed. Flexible residue A2974 of the 25S rRNA appears to support the tRNA interaction with the P loop by providing a physical obstacle that prevents the tRNA 3′-CCA end from moving back to the A loop. The NC is bound to tRNA via nucleotide A76, which is stabilized by A2822 and A2823 of the 25S rRNA. Overall, these five purine bases stabilize the 3′-CCA end in its canonical position on the P site, similar to the human post-translocational complex^22^. On the E site, the 3′-CCA end of the P/E tRNA is stabilized by interactions with 25S rRNA elements and with eL42 (Fig. 2b). A76 is tightly packed between Gm2796 and G2797 of the 25S rRNA, while C75 forms π-stacking with Tyr43 of eL42.

To assess potential species-specific differences, we inspected the functional interactions in the catalytic core of the tobacco ribosome and compared them with those of human 80S ribosomes in the same conformation: rotated with two bound tRNAs in hybrid positions^16^ (Fig. 2 and Supplementary Table 5). Overall, the plant ribosome interactions with both tRNAs are remarkably similar to those found in human 80S, which further supports the highly conserved functional core of the ribosome among eukaryotes. Notably, in our structure, the 3′-CCA tail of the A/P tRNA is nicely resolved and is virtually identical to a canonical P-site tRNA^22^. Presumably, the presence of the NC bound to the peptidyl-tRNA in our structure helps stabilize its tight interaction with the peptidyl transferase centre, thus allowing the visualization of this interaction.

### Role of metal ions in the decoding centre

**Fig. 3:**
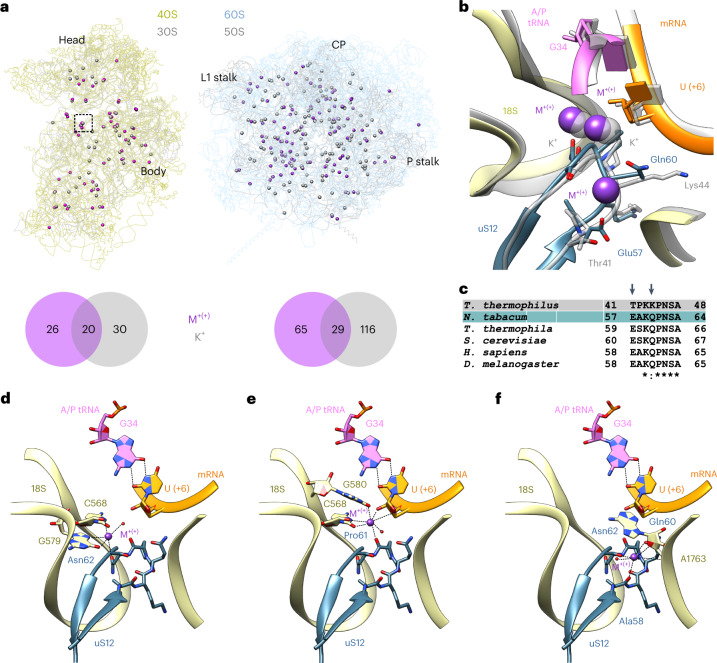
Putative potassium ion assignments in the tobacco 80S ribosome and their role in the decoding centre. **a**, Metal ions (M^+(+)^, purple; tentatively assigned potassium ions) found in the tobacco 40S (left, yellow) and 60S (right, blue) subunits in comparison with the potassium (K^+^) ions from the *T. thermophilus* 70S ribosome (PDB: 6qnr; underlaid in grey). The following structural landmarks are labelled: the head and body in the 40S subunit, and the CP, L1 stalk and P stalk in the 60S subunit. The Venn diagrams show the conservation of putative potassium binding sites in the tobacco (purple) and *T. thermophilus* (grey) small (left) and large (right) ribosomal subunits. **b**, Role of the putative potassium ions in the decoding centre (the boxed area in **a**) of the tobacco 80S ribosome. The overlay of the ribosome decoding centre from tobacco (in colour) and *T. thermophilus* (grey) shows the conservation of two potassium ion sites. The uS12 sequence difference results in a local charge difference that might explain the presence of the third putative potassium ion in the tobacco decoding centre. **c**, Alignment of the uS12 protein from several species shows the conservation of Glu57 and Gln60 in eukaryotes. **d**–**f**, Stabilization of the decoding centre by three putative K^+^ ions in the tobacco 40S subunit. For each K^+^ ion, the hydrogen bonds formed with neighbouring atoms are shown. The 18S rRNA is shown in yellow, uS12 is teal, A/P tRNA is pink, mRNA is orange, putative K^+^ ions are purple or white, and water molecules are shown as red spheres.

Overall, we found 140 putative K^+^ ions in the SSU and LSU of the tobacco 80S ribosome (Supplementary Fig. 7a). We subsequently compared their positions with the coordinates of the potassium ions in the 70S ribosome structure of *Thermus thermophilus*, as revealed by long-wavelength X-ray analysis^5^ (Fig. 3a). This comparison revealed both similarities and differences in the positions of potassium ions between 70S and 80S ribosomes.

For example, two K^+^ ions coordinate the stability of the decoding centre, with one of them directly supporting the codon–anticodon interaction in the translating 70S ribosome^5^. In the plant 80S structure, three putative K^+^ ions are found close to the decoding centre, stabilizing interactions between the mRNA, the uS12 protein and elements of the 18S rRNA, thus probably supporting the correct positioning of the codon in the ribosome (Fig. 3d–f). Two of these putative K^+^ ions occupy the same site as the K^+^ ions in the 70S ribosome of *T. thermophilus* (Fig. 3b). Interestingly, the uS12 sequence difference between *T. thermophilus* and tobacco leads to a change in a local charge distribution, which might explain the presence of the third metal ion in the decoding centre of the 80S ribosome (Fig. 3b,c). We found that residue Thr41 in *T. thermophilus* is replaced by Glu57 in tobacco, and residue Lys44 is replaced by Gln60. Both changes may facilitate the coordination of the K^+^ ion in tobacco (Fig. 3d).

### Chemical modifications of the plant 40S and 60S rRNA

**Fig. 4:**
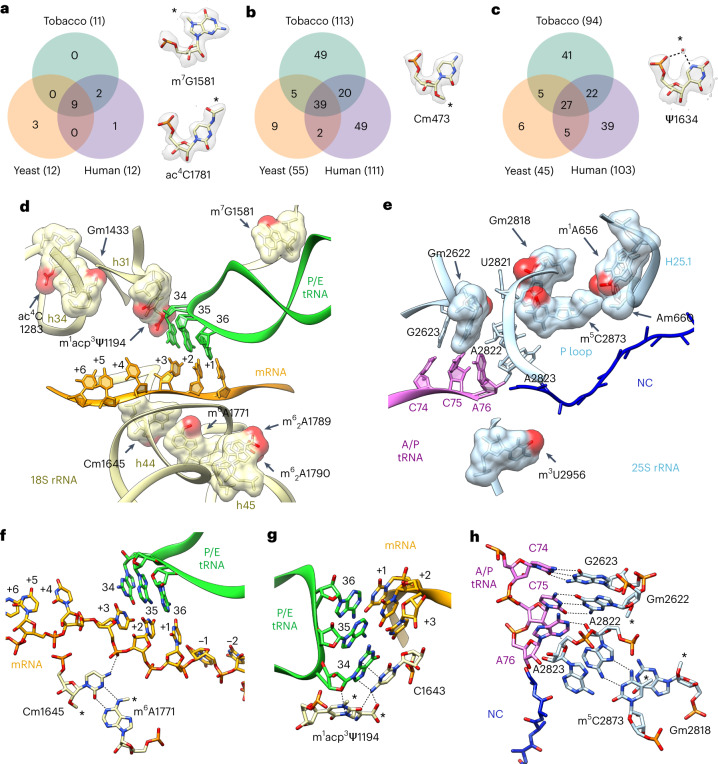
Conservation of chemical modifications of rRNA in eukaryotes and their role during translation. **a**–**c**, Venn diagrams showing the conservation of base modifications (**a**), 2′-O-Me sites (**b**) and Ψ sites (**c**) between tobacco, yeast and human rRNAs. Examples of the corresponding modifications (marked by the asterisks) are shown for each group of modifications. **d**,**e**, Accumulation of modified rRNA nucleotides near binding pockets of tRNAs. Panel **d** shows sites of 18S rRNA modification next to the codon–anticodon double helix formed by mRNA and P/E tRNA at the P site in the small ribosomal subunit. Panel **e** shows 25S rRNA methylation sites around the P site in the large ribosomal subunit next to the CCA tail of the A/P tRNA bound to the NC. Note that in all cases the added chemical groups (red) extend the interaction surface between the modified nucleotide and their neighbouring nucleotide(s). **f**–**h**, Molecular contacts of modified rRNA nucleotides with tRNAs and mRNA. Cm1645 directly contacts the mRNA codon and engages in base pairing with the N^6^-methyladenosine m^6^A1771 (**f**). m^1^acp^3^Ψ1194 interacts with C1643, and both residues stack with the ribose and base of G34 from the P/E tRNA (**g**). Base m^5^C2873 stacks with Gm2818 and base pairs with A2822, which in turn supports the proper positioning of A76 via A-minor interaction (**h**). Note that in all cases the added chemical groups (marked by asterisks) extend the interaction surface between the modified nucleotide and their neighbouring nucleotide(s). The 25S rRNA is shown in blue, the 18S rRNA is yellow, A/P tRNA is pink, P/E tRNA is green, mRNA is orange and the NC is dark blue.

The two most common modifications seen in the eukaryotic rRNA are 2′-OH ribose methylations (2′-O-Me) and pseudouridylation (Ψ)^7,8^. By visually inspecting our 2.2 Å resolution cryo-EM map and the quality of the model fit with regard to geometry and volume parameters, we found 113 2′-O-Me and 94 Ψ putative modification sites in the 18S, 25S and 5.8S rRNAs of the tobacco ribosome (Fig. 4b,c and Supplementary Fig. 10b,c). Comparison with the sites that had been previously biochemically identified in *Arabidopsis*^33–35^ and tomato (*S. lycopersicum*)^19^ revealed overall high similarity among the three species (Supplementary Fig. 10b,c and Supplementary Table 6) and supported our approach. The observed differences could reflect species- or tissue-specific modifications in the rRNA modification landscape or could be attributable to methodological limitations in either of the studies. Interestingly, we found several modification sites present in tomato and tobacco 80S (both belong to the Solanaceae family) but not in *Arabidopsis* (the Brassicaceae family). This finding indicates a species- and family-specific structural heterogeneity of the ribosomes at the level of chemical modifications. We further investigated the conservation of rRNA modifications between various eukaryotes (Fig. 4b,c and Supplementary Table 7). Overall, only about 30% of the 2′-O-Me and 94 Ψ sites are conserved among plants, yeast and mammals, while the vast majority of these modifications appear to be plant-specific.

Moreover, we found nine types of base modifications that had not been described in plants until very recently^19,36^ (Supplementary Fig. 10a). Most of these modifications are highly conserved across kingdoms (Fig. 4a) and are localized near functionally important sites of the SSU and LSU (Fig. 4d,e).

The elongating 80S ribosome structure presented here allowed us to directly inspect the role of chemical modification during translation. For example, nucleotide m^1^acp^3^Ψ1194 interacts with the anticodon stem–loop of the P-site-bound tRNA (Fig. 2d). This residue is adjacent to the mRNA–tRNA duplex and contacts the wobble base pair in the P site, probably stabilizing the 34 base rotation angle in the tRNA (Fig. 4g). Another modified nucleotide, Cm1645, directly contacts the mRNA codon at the +3 position. Interestingly, it forms a non-canonical base pairing with the nucleotide m^6^A1771, and apparently both modifications help maintain the proper position of the mRNA (Fig. 4f). Another region of the 80S ribosome that is enriched in modified nucleotides is the peptidyl transferase centre, located at the heart of the LSU. Three of the four base modifications in the 25S rRNA are localized close to the CCA end of the P-site tRNA. The modified nucleotide m^5^C2873 forms the non-canonical base pairing with the key nucleotide A2822, which in turn supports the proper positioning of the A76 nucleotide (Fig. 4e,h). The methyl groups of both m^5^C2873 and Gm2818 provide a larger surface for base stacking, while nucleotide U2821 is tightly sandwiched between the methyl groups of Gm2818 and Gm2622. Overall, these modifications seem to play a role in stabilizing the P loop. Finally, nucleotide m^3^U2956 is adjacent to the NC, forming the upper part of the tunnel wall (Fig. 4e).

### Intersubunit bridges in the rotated plant 80S ribosome

The present structure of the tobacco 80S ribosome enables the molecular assessment of interactions between the ribosomal subunits in the rotated-2 arrangement (Supplementary Fig. 11, top). Overall, the positions and components of the universal bridges (B1 to B8) in the tobacco 80S ribosome are highly conserved with those in the rotated configuration of the eukaryotic ribosomes from yeast^12^ and human^16,20^. In contrast, for the eukaryote-specific bridges (eB8, eB11, eB12 and eB13), which are located at the periphery of the ribosome, some specificities have been observed.

The solvent-exposed eukaryote-specific bridge eB13 is formed mainly with the help of protein eL24, which has a flexible linker wrapping around the side of the 40S body and a C-terminal helix reaching the back of the 40S subunit in yeast^12^ (Supplementary Fig. 14b,c). It has been suggested that the C-terminal helix of eL24 (in yeast, amino acids 87–128) strongly interacts with eS6 and h10 of the 18S rRNA regardless of intersubunit movements, and that the intersubunit movement is then facilitated via a flexible linker (in yeast, amino acids 59–80)^12,22^. By contrast, in the tobacco cryo-EM map, only the N-terminal domain of eL24 (amino acids 2–63), which is buried within the bottom of the 60S subunit, is structured, while the parts that are responsible for interaction with the components of the SSU (eS6, ES12^S^ and h10) are not visualized (Supplementary Figs. 14 and 15a–d).

**Extended Data Fig. 1:**
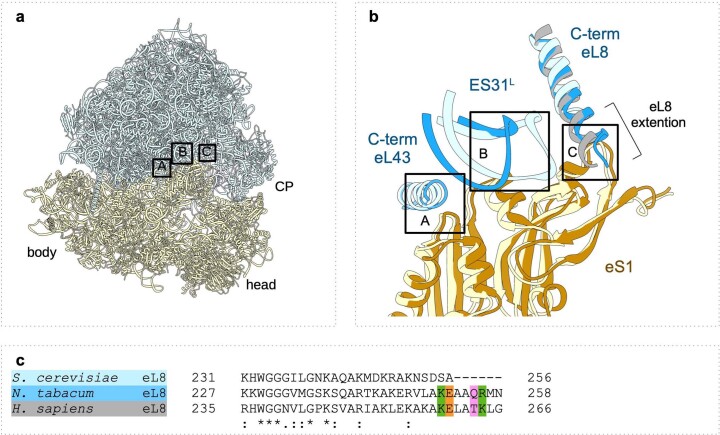
Comparison of the ribosomal eB8 bridge between different eukaryotes. (**a**) Interaction sites (A, B, C) between the 40S (yellow) and the 60S (blue) subunits representing bridge eB8 are mapped onto the 80S structure from tobacco. (**b**) 80S structures from tobacco (brown for the SSU and dark blue for the LSU), yeast (pdb: 4v88/ribosome B; yellow for the SSU and light blue for the LSU), human (pdb: 6y57; grey for the LSU) are aligned on the 60S subunit. Bridge eB8 is formed by interactions between eukaryote-specific eS1 on the SSU and eL43e (rectangular selection A) and eS31L (rectangular selection B) on the LSU in yeast. In tobacco and human 80S, another interaction with the eS1 protein is formed with help of the extended C-terminal end of eL8 (rectangular selection C). (**c**) Alignment of the C-termini of eL8 from *S. cerevisiae, N. tabacum*, and *H. sapiens* shows that tobacco and human ribosomes have an extended C-terminal end containing charged (highlighted green and orange) and polar (highlighted pink) amino acids.

**Extended Data Fig. 2:**
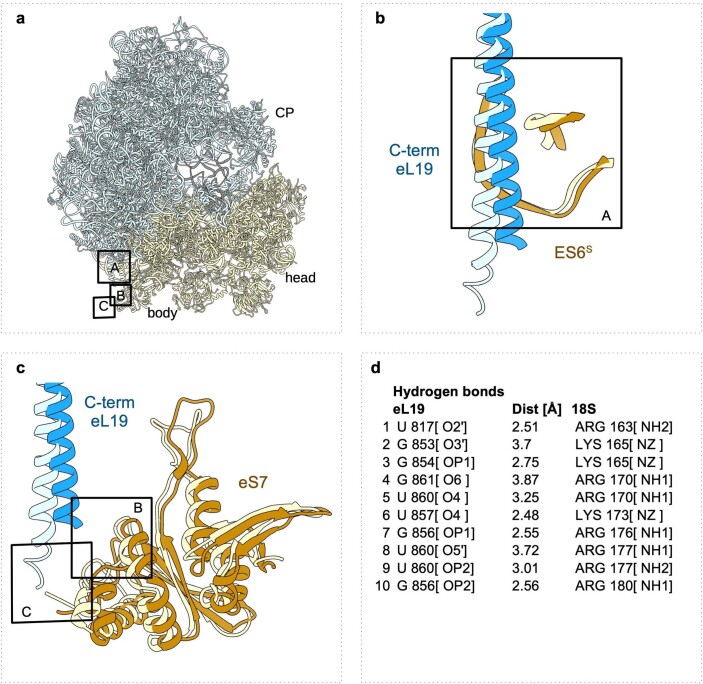
Comparison of the ribosomal eB12 bridge between different eukaryotes. **(a)** Interaction sites (A, B, C) between the 40S (yellow) and the 60S (blue) subunits representing the eB12 bridge are mapped onto the 80S structure from tobacco. In **(b)** and **(c)** 80S structures from tobacco (brown for the SSU and dark blue for the LSU) and yeast (pdb: 4v88/ribosome B; yellow for the SSU and light blue for the LSU) are aligned on the 60 S. **(b)** Bridge eB12 is formed by multiple hydrogen bonds between the C-terminal helix of the eukaryote-specific eL19 protein on the LSU and ES6^S^ on the SSU (rectangular selection A) in both tobacco and yeast. **(c)** In addition, the C-terminal helix of the eL19 interacts with the eukaryote-specific eS7, forming another interaction site (rectangular selection B in tobacco and rectangular selection C in yeast), which might vary depending on the rotational degree and thus position of the C-terminal residues of eL19. **(d)** Distances between atoms forming the hydrogen bonds in **(b)** as calculated by using the PISA tool^79^ (http://www.ebi.ac.uk/pdbe/prot_int/pistart.html).

**Extended Data Fig. 3:**
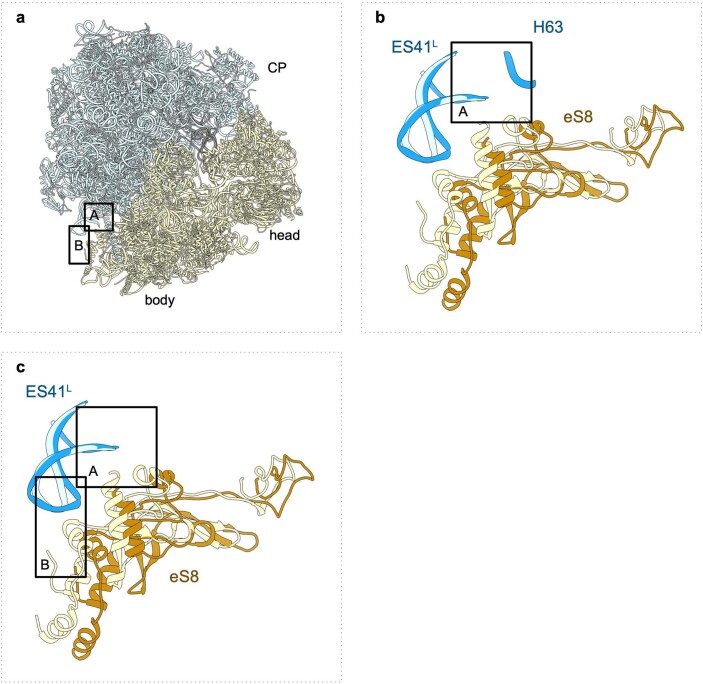
Comparison of the ribosomal eB11 bridge between different eukaryotes. **(a)** Interaction sites (A and B) between the 40S (yellow) and the 60S (blue) subunits representing the eB11 bridge are mapped within the 80S ribosome structure from tobacco. In **(b)** and **(c)** 80S structures from tobacco (brown for the SSU and dark blue for the LSU) and yeast (pdb: 4v88/ribosome B; yellow for the SSU and light blue for the LSU) are aligned on the 60S subunit. **(b)** In tobacco bridge eB11 is formed by interactions between the eukaryote-specific protein eS8 on the SSU and elements of ES41^L^ and H83 on the LSU (rectangular selection A). **(c)** In yeast bridge eB11 is formed by interactions between the eukaryote-specific protein eS8 on the SSU and ES41^L^ on the LSU (rectangular selections A and B). These differences in interaction sites reflect slightly different degrees of rotation and displacement of the eS8 protein relative to the LSU.

Comparison of sequences and secondary structural elements revealed a relatively moderate conservation of the eL24 protein between tobacco and yeast (Supplementary Fig. 15e). Nevertheless, according to the secondary-structure prediction, the two helices (α4 and α5) responsible for interaction with h10 and eS6 in the SSU in yeast are also present in tobacco eL24, although the unstructured distal C-terminal end of eL24 differs between tobacco and yeast (Supplementary Fig. 15e). In agreement with this finding, a direct interaction between plant eL24 and eS6 was not confirmed when examined in a yeast two-hybrid system^24^. Interestingly, a plant-specific reinitiation-supporting protein (RISP) was shown to interact with eL24 and eS6, thus ‘bridging’ plant 40S and 60S during the reinitiation of translation^24,37^. Remarkably, impressive conservation of the most distal part of the C-terminal end of eL24 (containing positively charged amino acid residues) was revealed in all vascular plants, suggesting that our model for the bridge eB13 represents a general feature for the green lineage (Supplementary Fig. 16). (The plasticity of eukaryote-specific bridges eB8, eB11 and eB12 is further discussed in the Supplementary Information and Extended Data Figs. 1–3.)

Recently, an 80S ribosome structure from tomato (*S. lycopersicum*) also became available^19^. In contrast to our purification strategy, the authors isolated the 80S fraction, which is apparently dominated by inactive 80S ribosomes without bound tRNAs. We compared both structures and found multiple local structural differences, which can be attributed to different physiological states. For example, in the tobacco ribosome, several structural changes in the A and P sites are found and are apparently required to accommodate bound tRNAs (Supplementary Fig. 12). Moreover, the SSU in the tobacco 80S structure is rotated by 10.6 degrees relative to the SSU in the tomato ribosome. The latter thus appears to be in the classical conformation (Supplementary Fig. 13a). We therefore compared all intersubunit bridges between the classical conformation and our rotated conformation. This analysis reveals large dynamics in the overall landscape of intersubunit interactions (Supplementary Fig. 11 and Supplementary Table 8), which is probably mediated by the structural changes (Supplementary Fig. 13b,c).

In summary, our data show that the formation and disruption of intersubunit bridges are accompanied by local structural rearrangements in the 80S ribosome and lead to the SSU rotation. Subsequently, this rotation results in tRNA translocation through the ribosome, thus driving translation elongation^20^.

### Plant-specific expansion segments and associated proteins

**Fig. 5:**
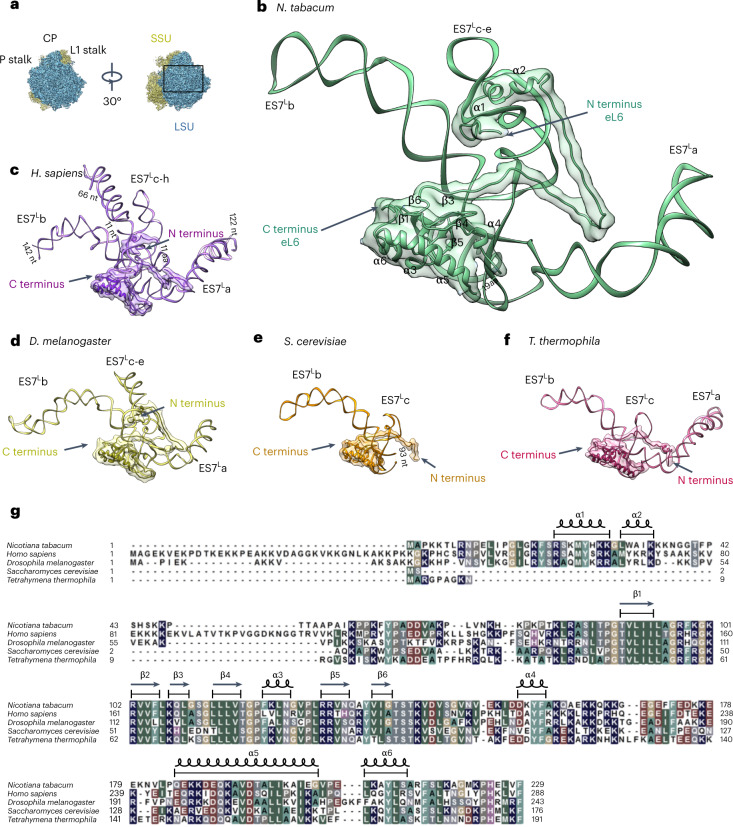
Suggested coevolution between the three-way junction of ES7Lc and the N terminus of eL6 in different representative eukaryotes. **a**, The position of ES7^L^ on the back side of the LSU is marked by a rectangle (expanded in **b**). The LSU is shown in blue; the SSU is shown in yellow. The CP, P stalk and L1 stalk are marked for orientation. **b**–**f**, Interactions of eL6 with ES7^L^ in ribosomes from different species: *N. tabacum* (nucleotides 424 to 647 of the 25S rRNA (representing ES7^L^) are shown) (**b**), *H. sapiens* (PDB: 6ek0; nucleotides 436 to 1311) (**c**), *D. melanogaster* (PDB: 4v6w; nucleotides 444 to 784) (**d**), *S. cerevisiae* (PDB: 5m1j; nucleotides 425 to 634) (**e**) and *T. thermophila* (PDB: 4v8p; nucleotides 422 to 658) (**f**). Ribosomal RNA and the eL6 protein are shown as ribbons. For clarity, simulated density maps filtered to a resolution of 7 Å are also shown for eL6. The branches of ES7^L^ and the N and C termini of eL6 are marked. For eL6 from *N. tabacum*, α-helices and β-sheets are indicated. aa, amino acids; nt, nucleotides. **g**, Sequence alignment of eL6 from five organisms. The secondary structure elements of eL6 from *N. tabacum* as derived from our structure in **b** are shown above the sequence. The background colours indicate conservation and highlight biophysical properties or specific amino acid side chains: blue indicates positively charged, brown indicates negatively charged, cyan/green indicates aromatic and hydrophobic, green indicates hydrophobic, grey indicates hydrophilic, dark grey indicates proline, and magenta indicates histidine.

The pronounced presence of plant-specific elements in the tobacco 80S ribosome is seen in the back of the LSU and associated with three expansion segments (ESs) of the 25S rRNA: ES7^L^, ES39^L^ and ES15^L^. Among these, ES7^L^ is one of the largest and most diverse ESs in the eukaryotic LSU. It consists of three main branches: ES7^L^a, stretching to the L1 stalk; ES7^L^b, expanding to the P stalk; and ES7^L^c (ES7^L^c-e in *Drosophila*^38^ and ES7^L^c-h in human^39^), extending to the central protuberance (CP) (Fig. 5). The branches of ES7 are stabilized on the surface of the ribosome via several eukaryote-specific proteins (for example, eL6 and eL28) or eukaryote-specific extensions of conserved proteins (for example, uL4 and uL30).

To assess kingdom-specific features, we compared the tobacco ribosome structure with four available high-resolution structures of eukaryotic ribosomes from different kingdoms (Supplementary Table 9). Plant ES7^L^ has an overall size similar to that of single-cell eukaryotes such as yeast^40^ and *Tetrahymena*^13^ (~220, ~210 and ~240 nucleotides, respectively). In *Drosophila*^38^ and especially in human^39^, its size is larger, comprising ~340 and ~860 nucleotides, respectively. The plant ES7^L^c helix splits into two additional branches (ES7^L^d and e)^18^, forming a three-way junction stabilized by the N-terminal end of the eukaryote-specific eL6 protein, similar to human and *Drosophila* (Fig. 5b–d). By contrast, both yeast and *Tetrahymena* lack the three-way junction of the ES7^L^c helix, and the N-terminal extension of eL6 is absent from ribosomes of both organisms (Fig. 5e,f).

The globular C-terminal domain of eL6 is highly conserved in both sequence and structure among five representative organisms (Fig. 5g). Notably, it has an insertion between the two terminal α-helices (α4 and α5) in tobacco, which is mostly unstructured. This insertion contains many charged amino acids such as Lys, Arg, Glu and Asp and forms a loop that interacts with the branch of ES7^L^a. Protein eL6 also contains an N-terminal stretch that varies markedly among the analysed structures. In three of them (tobacco, human and *Drosophila*), it has a similar fold: an α-helix (α1 and α2 in tobacco, Fig. 5b) passing through a three-way junction of ES7^L^c. In the other structures (yeast and *Tetrahymena*), the N-terminal stretch of eL6 is shorter and only reaches the basal part of ES7^L^a.

In tobacco, several amino acids facilitate the interaction between the N-terminal end of eL6 and ES7^L^c-e, similar to human and *Drosophila*. Interestingly, the region corresponding to the α1- and α2-helices has high sequence similarity in all three organisms (Fig. 5e). We performed an extended sequence analysis to find out whether this RNA–protein interaction might be preserved among eukaryotes and whether this region is conserved in other species from the plant kingdom (Supplementary Fig. 17). This analysis revealed that the eukaryote-specific eL6 diverges markedly in sequence and length among the examined organisms, with the phylogenetic analysis clearly separating all the species into distinct phyla and divisions (Supplementary Fig. 17a). Only the globular C-terminal domain of eL6 composed of β-sheets appears to be highly conserved among the analysed species. By contrast, the N-terminal stretch of eL6 is the least preserved part among eukaryotes. However, in members of the plant kingdom, this N-terminal stretch exhibits a higher level of conservation (Supplementary Fig. 17b,c). It is therefore likely that the structure and specific interactions between the N-terminal end of the eL6 protein and ES7^L^c-e overall are preserved in the green lineage, from the unicellular green alga *Chlamydomonas reinhardtii* to seed plants. Moreover, this N-terminal extension of eL6 seems to be present only among members of the plant and animal kingdoms; in the analysed fungi and protists, this part is absent.

**Extended Data Fig. 4:**
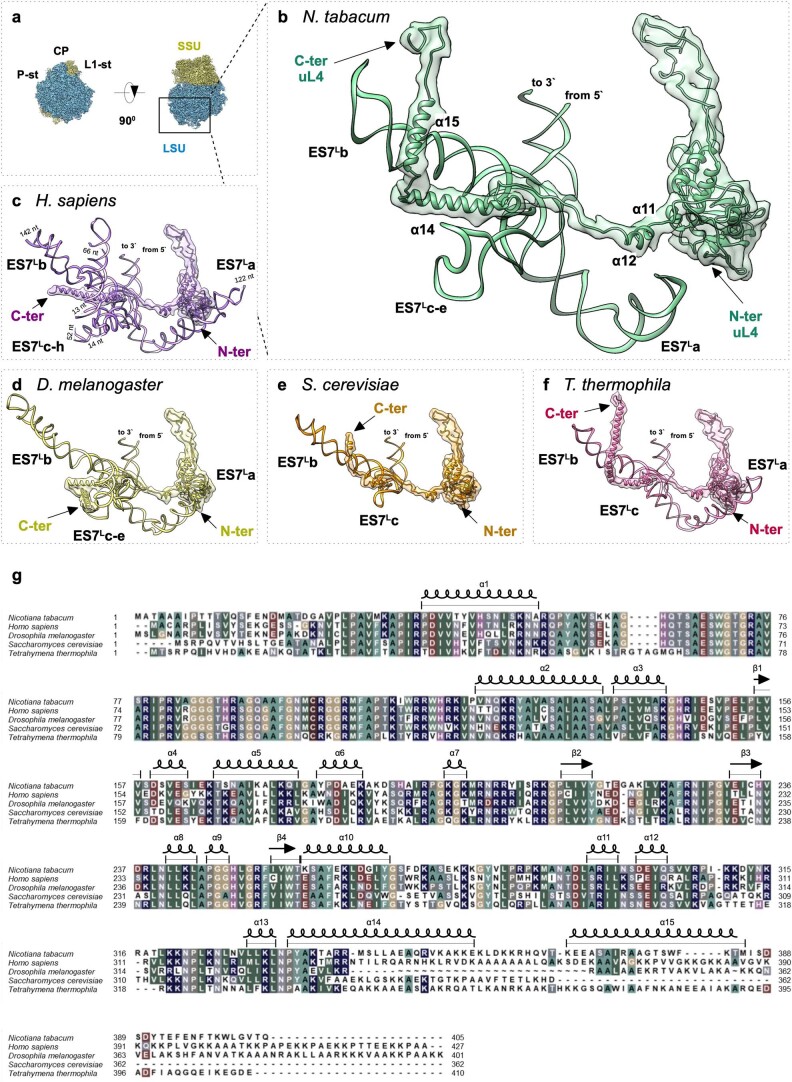
Interaction between ES7^L^c and the C-terminus of uL4 in different representative eukaryotes. **(a)** The position of ES7^L^ on the back side of the large subunit is marked by a rectangle. Large subunit, LSU (blue). Small subunit, SSU (yellow). Central protuberance (CP), P-stalk (P-st), and L1-stalk (L1-st) are marked. **(b-f)** Interactions of the eukaryote-specific C-terminus of uL4 with ES7^L^ in ribosomes of different species: **(b)** *N. tabacum* (nucleotides from 424 to 647 of the 25S rRNA representing ES7^L^ are shown), **(c)** *H. sapiens* (pdb: 6ek0; nucleotides from 436 to 1311), **(d)** *D. melanogaster* (pdb: 4v6w; nucleotides from 444 to 784), **(e)** *S. cerevisiae* (pdb: 5m1j; nucleotides from 425 to 634), and **(f)** *T. thermophila* (pdb: 4v8p; nucleotides from 422 to 658). Ribosomal rRNA and uL4 protein are shown as ribbons. Simulated density maps for protein uL4 filtered to a resolution of 7 Å are shown for better orientation. Branches of ES7^L^ (a–h) and the N- and C-termini of uL4 are marked. For uL4 from *N. tabacum*, selected α-helices are indicated. **(g)** Sequence alignment of uL4 from five organisms. Secondary structure elements of uL4 from *N. tabacum* are shown above the sequences and are derived from the structure shown in **(a)**. Specific background colours indicating conservation and highlighting specificities or specific amino acid side chains are as in Fig. 5.

**Extended Data Fig. 5:**
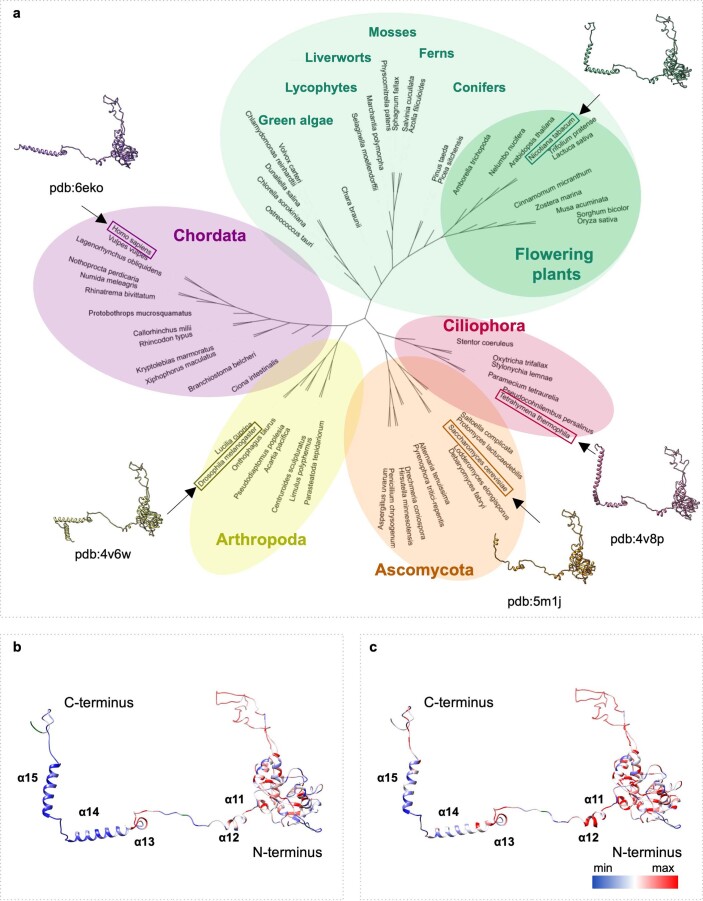
Sequence conservation of the uL4 protein. **(a)** Phylogenetic tree based on uL4 amino acid sequences from various organisms. Pink: phylum Ciliophora (with representative structure from *T. thermophila*) from the protist kingdom. Purple and yellow: phylum Chordata (*H. sapiens*) and phylum Arthropoda (*D. melanogaster*), both from the animal kingdom. Orange: phylum Ascomycota (*S. cerevisiae*) from the fungi kingdom. Dark green: division (phylum) Magnoliophyta (flowering plants; *N. tabacum*) from the plant kingdom. Light green: other divisions of plants. Species for which a structure is available are marked by a rectangle. Corresponding structures are shown for selected phyla. In **(b)** and **(c)** uL4 sequence conservation over all species and in plants was mapped to the protein structure. Conservation level of each amino acid is mapped into the uL4 structure from *N. tabacum*. The colour bar shows a gradient from minimal (blue) to maximal (red) conservation level among selected organisms. Selected α-helices and β-sheets are indicated, and C- and N-termini are labelled.

**Extended Data Fig. 6:**
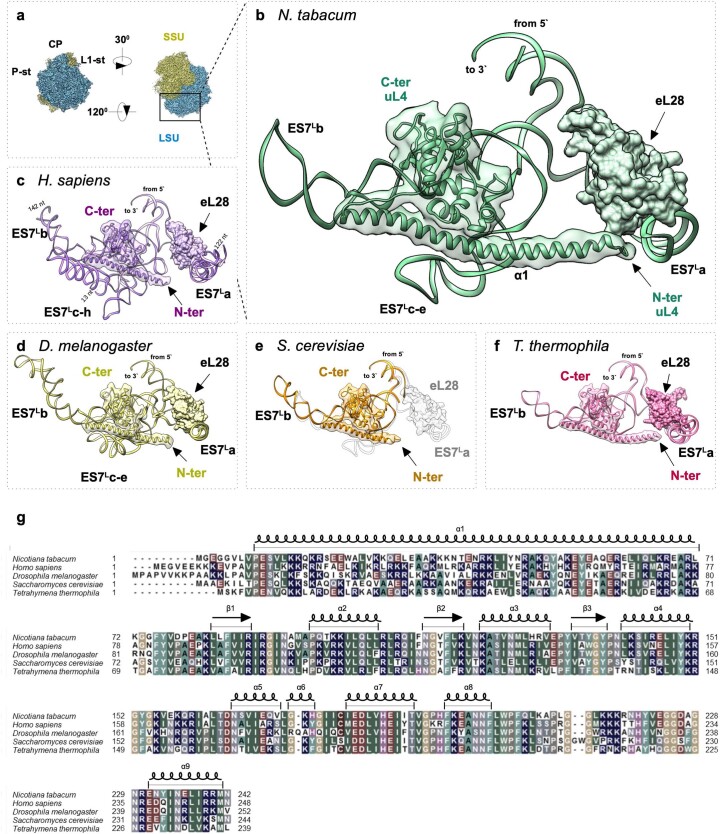
Interaction between ES7^L^a, eL28 and uL30 in different eukaryotes. **(a)** The position of ES7^L^ on the back side of the large subunit is marked by a rectangle. Large subunit, LSU (blue). Small subunit, SSU (yellow). Central protuberance (CP), P-stalk (P-st), and L1-stalk (L1-st) are marked for orientation. **(b-f)** Interactions of the eukaryote-specific N-terminus of uL30 with ES7^L^ in the ribosome from different species: **(b)** *N. tabacum* (nucleotides from 424 to 647 of the 25S rRNA, representing ES7^L^), **(c)** *H. sapiens* (pdb: 6ek0; nucleotides from 436 to 1311), **(d)** *D. melanogaster* (pdb: 4v6w; nucleotides from 444 to 784), **(e)** *S. cerevisiae* (pdb: 5m1j; nucleotides from 425 to 634), and **(f)** *T. thermophila* (pdb: 4v8p; nucleotides from 422 to 658). Ribosomal rRNA and uL30 protein are shown as ribbons. Simulated density maps for protein uL4 filtered to a resolution of 7 Å are underlaid. Protein eL28 is shown as surface. Branches of ES7^L^ (a–h) and the N- and C-termini of uL30 are marked. For uL30 from *N. tabacum*, α-helix α1 is indicated. **(g)** Sequence alignment of uL30 from five organisms. The secondary structure elements of uL30 from *N. tabacum* are shown above its sequence and are derived from the structure shown in **(a)**. Specific background colours indicate conservation and highlight specificities or specific amino acid side chains (details as in Fig. 5).

**Extended Data Fig. 7:**
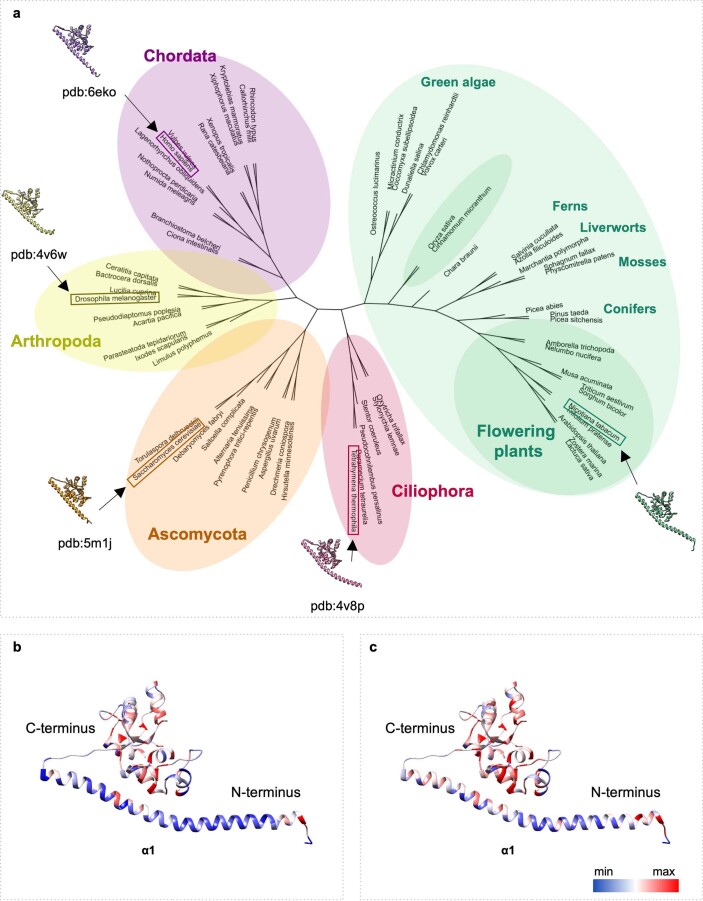
Sequence conservation of the uL30 protein. **(a)** Phylogenetic tree based on uL30 amino acid sequences from various organisms. Pink: phylum Ciliophora (with representative structure from *T. thermophila*) from the protist kingdom. Purple and yellow: phylum Chordata (*H. sapiens*) and phylum Arthropoda (*D. melanogaster*), both from the animal kingdom. Orange: phylum Ascomycota (*S. cerevisiae*) from the fungi kingdom. Dark green: division (phylum) Magnoliophyta (flowering plants; *N. tabacum*) from the plant kingdom. Light green: other divisions of plants. Species for which a structure is available are marked by a rectangle. Corresponding structures are shown for selected phyla. **(b)** and **(c)** uL30 sequence conservation over all species and in plants was mapped to the protein structure. Conservation level of each amino acid is mapped into the uL30 structure from *N. tabacum*. The colour bar shows a gradient from minimal (blue) to maximal (red) conservation level among selected organisms. Selected α-helices and β-sheets are indicated, and C- and N-termini are labelled.

Additional proteins associated with ES7^L^ are eL28, uL4 and uL30. Although uL4 and uL30 are universally conserved RPs, they have evolved eukaryote-specific elements (Supplementary Fig. 18), which appear to interact with branches of ES7^L^. The role of these proteins in stabilizing ES7 as well as their conservation among various eukaryotes is further discussed in the Supplementary Information and Extended Data Figs. 4–7.

The present study shows that ES7^L^ in the tobacco 80S ribosome has a unique architecture, deviating from the structures in other eukaryotic ribosomes. Eukaryote-specific RPs as well as eukaryote-specific protein extensions and insertions were found to be associated with ES7^L^ and show a large level of sequence variability. At the same time, we observed substantial conservation of secondary structural elements, resulting in similar modes of interaction with rRNA elements (Fig. 5 and Extended Data Figs. 4 and 6). Altogether, these findings suggest that eukaryote-specific elements of the 80S ribosome—namely, ESs and eukaryote-specific RPs—have coevolved. The result of this coevolution seems to be an overall similar (but not identical) architecture and interaction network on the outer eukaryote-specific layer of the 80S ribosome. In agreement with this idea, the tobacco 80S ribosome shows a pronounced level of kingdom-specific features on its surface. Overall, our phylogenetic analyses, which included organisms from diverse groups of photosynthetic eukaryotes, reveal that most structural features discovered in the tobacco ribosome are well conserved in the whole plant kingdom (Supplementary Fig. 17 and Extended Data Figs. 5 and 7).

## Discussion

Our cryo-EM structure for the *N. tabacum* 80S ribosome with an overall resolution of 2.2 Å made it possible to determine the atomic coordinates of rRNAs and RPs in unprecedented detail. Importantly, our ribosome structure was captured in an active state and includes two bound tRNAs, mRNA and an NC passing through the exit tunnel, thus revealing new molecular details of the translation process in plant cells. Using polysomes as the starting material for sample preparation allowed us to obtain the tobacco 80S structure in a different physiological and conformational state from the recently reported 80S ribosome from tomato^19^. Both structures come from species of the Solanaceae family and are in general similar, although it was possible to resolve two proteins (eS12 and eS31) localized in the head of the 40S subunit of the tobacco ribosome that are missing in the tomato structure. Moreover, our findings reveal multiple local structural differences, attributed to the presence or absence of bound ligands as well as a different degree of 40S subunit rotation. We believe that the two independent approaches to resolve plant ribosomes are complementary. They are helpful on one hand to validate each other; on the other hand, they directly reveal differences between different physiological states in plant ribosomes.

Comprehensive analysis of the rRNAs in the tobacco 80S ribosome revealed overall more than 200 nucleotide modifications of 14 different types, 9 of which had not been shown for the plant ribosome until very recently^19^. The comparison of rRNA modification patterns in the tobacco 80S ribosome with those in human and yeast revealed a remarkably high degree of kingdom specificity in the modified sites by identifying 80 plant-specific modification sites. The high quality of the map allowed us to directly assess how rRNA modifications coordinate and stabilize the positioning of tRNAs and mRNA in their binding pockets in the ribosome during protein synthesis. For example, our map reveals how chemical modifications stabilize the codon–anticodon interaction in the P site of the 40S subunit during initiation and the binding of the tRNA CCA end at the P loop of the 60S subunit during peptide bond formation.

Furthermore, Mg^2+^ ions and monovalent cations such as K^+^, the polyamines spermine and spermidine, and water molecules have been successfully mapped within the tobacco 80S ribosome structure. We were able to reveal eukaryote-specific features of the SSU by localizing monovalent cations in the decoding centre and comparing their positions with those in the 70S structure from *T. thermophilus*.

The reconstruction of the 80S ribosome from plants also allowed us to perform a systematic comparative structural and phylogenetic analysis in eukaryotes. This analysis revealed that the inner functional core of the ribosome, which includes the binding sites for the tRNA molecules, is remarkably conserved among eukaryotes. By contrast, the outer eukaryote-specific layer^41^ of the 80S ribosome exhibits a high level of kingdom-specific plasticity. For example, our structure does not include a density for the C-terminal part of the eL24 protein, which, together with several elements in the SSU (including the eS6 protein), forms the eB13 bridge in yeast and human. It was previously shown that the C-terminal part of eL24 from *Arabidopsis* interacts with the plant-specific RISP, which also binds to eukaryotic initiation factor 3 and eS6 (refs. ^24,37^). It has been proposed that, depending on its phosphorylation status, RISP bound to the C terminus of eS6 may subsequently capture the 60S subunit via its interaction with the C terminus of eL24. This suggests a RISP function in translation reinitiation of polycistronic mRNAs^42^. In light of our structure, these data suggest that, in contrast to yeast and human, eL24 might have an additional role during translation in plants, and the interaction between eL24 and eS6 requires the presence of the plant-specific RISP factor, which potentially regulates reinitiation.

Another plant-specific region of the 80S ribosome is ES7^L^, which is localized close to the NC exit tunnel. A few studies in yeast have reported a role of ES7^L^ in the recruitment of factors involved in co-translational protein modification^43^. Interestingly, the structure of the plant N-terminal acetyltransferase complex C (NatC) seems to deviate notably from its yeast counterpart^44^. Presumably to support eukaryote-specific processes such as co-translational polypeptide modification, the interaction between NatC and the ribosomal surface near the NC tunnel has been shaped by coevolution. Linked evolution of RPs and ribosome-associated proteins would result in structural deviations but conserved enzymatic activity in phylogenetically distant organisms.

In summary, our study uncovers numerous plant-specific structural features of the 80S ribosome that may reflect specific mechanisms of translational regulation. Our atomic model of the plant 80S ribosome provides a solid molecular basis for future structural and functional studies of translation and its regulation in plant cells.

## Methods

### Plant material and purification of plant polysomes

Wild-type tobacco plants (*Nicotiana tabacum cv. Petit Havana*) were grown in a greenhouse under physiological conditions (at 24 °C for 21 days). Aerial parts were harvested five hours after the onset of light, when plant translation is fully active^45,46^. Immediately after harvesting, the tissue was snap-frozen in liquid nitrogen to freeze cellular processes including translation. For ribosome isolation, tobacco leaves were ground in a mortar, and an extraction buffer containing 40 mM HEPES/KOH pH 8.0, 100 mM KCl, 15 mM MgCl_2_, 25 mM EGTA, 0.5 mg ml^−1^ heparin, 0.5 mM spermidine, 0.04 mM spermine and 10 mM DTT was added. The extraction buffer also contained 1% (w/v) Triton-100 and 2% (w/v) polyoxyethylene and was supplemented with protease and RNase inhibitors. Subsequently, the lysate was loaded onto a sucrose cushion (54% (w/v) sucrose in extraction buffer excluding detergents) to enrich large macromolecular complexes including polysomes by ultracentrifugation for three hours at 50,000 *g*^47^. After this step, the pelleted polysomes were resuspended in a sucrose-free, detergent-free buffer containing 40 mM HEPES/KOH pH 8.0, 100 mM KCl, 15 mM MgCl_2_, 25 mM EGTA, 0.5 mM spermidine, 0.04 mM spermine and 1 mM DTT, and the sample was directly used for the preparation of cryo-EM grids. All the steps were carried out at 4 °C.

### Cryo-EM grid preparation

For cryo-EM, 300 mesh R3/3 Cu grids from Quantifoil Micro Tools with an additional 2 nm amorphous carbon support layer on top were freshly glow discharged in a Fishione Model 1070 Nano Clean plasma cleaner for 1 min with a gas composition of 95% argon and 5% oxygen and a forward power of 30%. Then, 3.5 µl of freshly prepared sample was applied onto the grids and incubated for 45 s. The grids were plunge-frozen in liquid ethane using a Vitrobot Mark IV with the following settings: 4 °C, 100% humidity, blot force 0, blotting time 2 and 4 s.

### Cryo-EM data acquisition

To check the sample quality, a small dataset was collected on a 120 kV Tecnai Spirit electron microscope with a total dose of 30 e Å^−2^ using a F416 TemCam CMOS based 4k × 4k detector (TVIPS) at a nominal magnification of ×42,000 (yielding a pixel size 2.65 Å). In total, approximately 400 images were obtained. The dataset was collected at defocus values ranging from −1 to −2.5 µm.

For the high-resolution high-throughput microscopy, framesets were collected on a Titan Krios G3i transmission electron microscope (ThermoFisher Scientific, Server Version 2.15.3, TIA Version 5.0) equipped with an extra-bright field-emission gun, a BioQuantum post-column energy filter (Gatan) and a K3 direct electron detector (Gatan Digital Micrograph Version 3.32.2403.0). The images were recorded at an acceleration voltage of 300 kV in low-dose mode as dose-fractionated videos using EPU Version 2.8.1 (ThermoFischer Scientific) with a maximum image shift of 5 µM using aberration-free image shift. In total, 14,651 videos with a total dose of 27 e Å^−2^ each split over 27 fractions (with an individual dose of 1 e Å^−2^ per fraction) were recorded in energy filtered zeroloss (Slit Width 20 eV), nano-probe mode at a nominal magnification of ×81,000 (resulting in a calibrated pixel size of 0.53 Å on the specimen level) in super-resolution mode with a 100 µM objective aperture. Data were collected with defocus values ranging from −0.5 to −1.8 µm on Quantifoil R2/2 300 Mesh Grids. In each hole, seven exposures were acquired.

### Cryo-EM image processing

The collected framesets were corrected for local movement with patch alignment and were dose weighted using MotionCor2 (ref. ^48^). All 27 frames were used for the resulting dose-weighted micrograph. The contrast transfer function parameters were estimated using Gctf^49^, and particles were picked inside the SCIPION program^50^ using Gautomatch in reference-free mode.

Particles were extracted, normalized and binned twofold with RELION^51^, yielding a particle stack with a pixel size of 1.06 Å. The particle stack was subjected to 2D classification in cryoSPARC^52^, and particles corresponding to artefactual classes were removed from the dataset. The remaining particles, consisting of a mixture of 70S and 80S particles, were aligned onto a common reference obtained by ab initio reconstruction in cryoSPARC.

To separate 70S particles from the cell organelles (mainly chloroplasts) and 80S particles from the cytosol, a multireference sorting approach^22,26^ was performed using SPIDER software^53^. Particle orientations were converted to SPIDER format using custom Python scripts and briefly equilibrated in a local refinement to account for differences in the frame of reference of the software packages, before performing a 3D classification. During multiparticle 3D refinement, the data were binned sixfold for the initial sorting (corresponding to tiers 1 and 2 in the sorting tree; pixel size, 3.18 Å; Supplementary Fig. 2), while further sorting (tier 3 in the sorting tree) was performed with fourfold decimated data for finer details (pixel size, 2.12 Å). To guide the sorting procedure, 3D variability analysis^22^ was performed at various stages, and masks for focused reassignment were generated from the resulting heterogeneity using SPIDER.

After rigorous sorting, particles representing the largest homogeneous physiological state (rotated-2) were isolated and used to reconstruct a high-resolution structure using data at pixel size 0.848 Å. To improve the reconstruction, particles were subjected to a per-particle contrast transfer function refinement in RELION v.3.1 (ref. ^54^). The resulting 80S ribosome reconstruction was further refined using the non-uniform refinement in cryoSPARC, including the optimization of higher-order aberrations. The resulting volume was used as input for separate local refinements of the 60S and 40S subunits in cryoSPARC.

For the local refinement of the subunits, soft masks were created by first manually segmenting the density maps using the Segger watershed algorithm implemented in Chimera^56^. The individual densities for the LSU and SSU were then extracted, thresholds were set to remove noise and negative densities, the remaining densities were dilated for three steps, and a cosine edge of nine steps was applied to the edges of the nascent masks. All these steps were performed using a custom procedure implemented in SPIDER. After conversion to MRC format, the masks were then imported into cryoSPARC, and local refinement was applied.

Since the local refinements gave very modest improvements in resolution due to the high homogeneity of the state, the processing was terminated at this point. A final reconstruction was obtained using cryoSPARC with standard B-factor sharpening, and the maps were filtered to a local resolution before interpretation.

Gold-standard Fourier shell correlation curves and cross-resolution curves between the experimental map and density simulated from the model were calculated in SPIDER using soft masks.

### Model building and refinement

The structure presented here was solved at near-atomic resolution, allowing unambiguous model building with Coot^55^. The reference models of the yeast rRNAs (PDB: 5m1j; ref. ^40^), yeast and human RPs (PDB: 6ek0; ref. ^39^) and human tRNAs (PDB: 5aj0; ref. ^22^) were placed into the density using the rigid-body docking function in UCSF Chimera^56^.

The sequences of *N. tabacum* 5S, 5.8S, 18S and 25S rRNA as well as tRNA used to build the model were obtained through the SGN database^27^, using the corresponding sequences from *Arabidopsis* as templates (Supplementary Table 2). The current structure contains a mixture of all endogenous tRNAs present in actively translating 80S ribosomes from the plant cytosol. To build atomic coordinates for tRNAs occupying the A, P and E sites on both ribosomal subunits, a sequence for the Phe-tRNA was taken (Supplementary Table 2). The RNA sequences were mutated into the tobacco counterparts in Coot and then visually inspected and manually adjusted where residues did not fit well into the density. When the quality of the map allowed, the structures of the plant-specific rRNA parts (including ESs) were manually built using Coot with the aid of the secondary structures that were predicted by the mfold web server^57^. Considering base stacking, base pairing and real-space fit into the experimental electron map, the RNA was globally idealized using ERRASER^58^. The models for mRNA and the NC were adapted from the mRNA model and NC model in a human classical POST ribosome structure (PDB: 5aj0; ref. ^22^).

The sequences of *N. tabacum* RPs were obtained from the National Center for Biotechnology Information (NCBI) protein database and are based on the list of proteins identified by MS (see below; Supplementary Tables 3 and 4). Tobacco RPs were modelled using a homology modelling tool of the SWISS-MODEL server^59^, and they were subsequently visually inspected in Coot and manually adjusted where amino acid residues did not fit well into the density. Plant-specific regions were built de novo, whenever the quality of the map allowed.

After RNA and protein residues had been modelled, the difference map was inspected, and the vast majority of the residual density was assigned to a ribosome solvation shell. We used a cryo-EM map filtered to a local resolution (calculated in cryoSPARC) to reduce noise and thus minimize bias when assigning residual density to ions and water molecules. Ribosome solvation (including water molecules, metal ions, and the polyamines spermine and spermidine) was modelled using a combination of manual inspection in Coot and PHENIX (phenix.douse)^60^. Spermine and spermidine molecules were modelled first, as they are easily distinguishable due to their elongated shape. The automated algorithm phenix.douse (part of PHENIX) was then run to identify and model water molecules. After this step, the remaining cryo-EM density was manually checked in Coot. The assignment of the metal ions—that is, magnesium (Mg^2+^), potassium (K^+^) or sodium (Na^+^)—within cryo-EM structure remains challenging. However, taking into account that Mg^2+^ has a strong preference for octahedral coordination (coordination number 6)^61,62^, the vast majority of Mg^2+^ ions could be identified unambiguously (Supplementary Figs. 6c and 7b). The assignment of other metal ions (K^+^ or Na^+^) within a cryo-EM structure is not trivial, though the monovalent and bivalent metal ions contribute substantially to the stability of rRNA structures^5^. On the basis of the buffer composition used for sample preparation, we attempted to identify coordinates for K^+^ ions. K^+^ ions are larger and have higher coordination numbers (8–12) and different geometry parameters than Mg^2+^. Using those criteria, we assigned all putative potassium ions (Supplementary Fig. 7a). The numbers of various solvent molecules are given in Supplementary Table 1. Afterwards, models for the 60S and 40S subunits were refined separately into their respective locally refined maps with PHENIX real-space refinement^63^, using maps filtered to the local resolution wherever needed to interpret regions of lower resolution.

Along with the individual models for the 60S and 40S, a full model for the 80S with bound tRNA, mRNA and NC was also built and refined into the 80S map obtained after a non-uniform refinement in cryoSPARC. This model was used to assess the molecular details of the RNA binding pockets for the two hybrid tRNAs. The final models were validated by using PHENIX and MolProbity^64^. The refinement statistics are given in Supplementary Table 1.

### Assignment of rRNA modifications

Chemical modifications were assigned by visual inspection of the cryo-EM maps, which were filtered to a local resolution in cryoSPARC. For smoothness, the maps were also supersampled in Coot prior to analysis.

After all the rRNA residues were modelled, each nucleotide was checked for the presence of pronounced additional densities in the ribosome 2′-OH position and on nucleotide bases. Most types of rRNA modification result from the addition of one or more chemical groups (for example, methyl, acetyl or carboxypropyl) to a nucleotide (Supplementary Fig. 9, indicated by asterisks). The corresponding rRNA modifications were identified using the quality of the fit with regard to geometry and volume parameters.

Pseudouridylation is harder to confirm structurally due to the isomeric nature of this modification. However, it forms characteristic hydrogen bonding in the N1 position, in most cases with the neighbouring water molecule. For each uridine, the geometric parameters for the presence of a characteristic hydrogen bond between the N1 position and a potential binding partner were manually checked, and if possible, a pseudouridine was modelled (Supplementary Fig. 8, indicated by dashed lines and asterisks).

Taking into account that the resolution is not constant throughout the map, each apparent modification was examined separately with the map contour level adjusted individually. Moreover, the presence or absence of nucleotide modifications was cross-checked by comparing its corresponding density with the neighbouring residues in their local highly isotropic environment.

### Sequence analysis of eL6, uL4 and uL30

Sequence analysis of eL6, uL4 and uL30, associated with ES7 on the 60S subunit, was performed for species representing phylogenetically distant groups of eukaryotes: phylum Ciliophora (*T. thermophila* 40S and 60S subunit structures) from the protist kingdom, phylum Ascomycota (*S. cerevisiae* 80S structure) from the fungi kingdom, and phylum Chordata (*H. sapiens* 80S structure) and phylum Arthropoda (*D. melanogaster* 80S structure), both from the animal kingdom, as well as various divisions (phyla) from the plant kingdom (*N. tabacum* 80S structure, Supplementary Table 10).

Using a protein–protein BLAST search^65^, we collected the sequences using several sources. For the vast majority of organisms, sequences were obtained from the NCBI protein database. For several algae, mosses, ferns and gymnosperms, sequences were retrieved from the following databases: Phytozome^66^ (https://phytozome.jgi.doe.gov), FernBase^67^ (https://www.fernbase.org) and ConGenIE^68^ (http://congenie.org) (Supplementary Table 10).

### Sequence alignments and phylogenetic tree analyses

First, the collected amino acid sequences of selected proteins (uL4, eL6 and uL30, identified by BLAST search) in the respective species were aligned with ClustalX (v.2.0.9)^69^. Second, all plant sequences of each domain were separately aligned with ClustalX (using the following multiple sequence alignment parameters: BLOSUM matrix; gap opening, 10; gap extension, 0.2; iteration of each alignment step). Sequence alignment presentations were performed with BioEdit^70^. Specific background colours indicate conservation (Blosum62 matrix) and highlight specificities or specific amino acid side chains. The resulting alignments (in FASTA format) were used by Molecular Evolutionary Genetics Analysis (Mega X)^71^ for phylogenetic analyses by maximum likelihood methods (the Jones–Taylor–Thornton model). The resulting trees were visualized with iTOL^72^ (https://itol.embl.de/).

### Sequence analysis and secondary structure elements of eL24

Sequences for eL24 protein were collected using a protein–protein BLAST search for various groups of plants from the databases specified in Supplementary Table 10. To address eL24 sequence conservation among plants, we performed multiple sequence alignment using Clustal Omega^73^ (https://www.ebi.ac.uk/Tools/msa/clustalo/). For the secondary structure prediction of the eL24 protein, we used the PSIPRED secondary structure prediction method implemented in the PSIPRED server (http://bioinf.cs.ucl.ac.uk/psipred/)^74,75^.

### MS analysis

To identify the 80S proteome of our ex vivo derived ribosomes, MS analysis was performed using an aliquot of the exact same sample used for cryo-EM. The samples were digested using a mixture of trypsin and LysC according to Glatter et al.^76^. Peptides were purified using Ziptips (Millipore) according to the manufacturer’s instructions. The peptides were resuspended in 5% (v/v) acetonitrile and 0.1% (v/v) formic acid and were then separated on a C18 reversed-phase analytical column (Acclaim PepMap100, Thermo Fisher Scientific) using an Easy-nLC 1000 liquid chromatography system (Thermo Fisher Scientific). The peptides were eluted using a nonlinear 5–34% acetonitrile gradient in 0.1% formic acid and 5% DMSO at a flow of 300 nl min^−1^. The gradient lasted 28 min. After the gradient, the column was cleaned for 10 min with 85% acetonitrile in 0.1% formic acid and 5% DMSO. Eluted peptides were transferred to an NSI source and sprayed into an Orbitrap Q-Exactive Plus mass spectrometer (Thermo Fisher Scientific). The MS was run in positive ion mode. For full MS scans, the following settings were used: resolution, 70,000; AGC target, 3E6; maximum injection time, 100 ms; scan range, 200 to 2000 *m*/*z*. For dd-MS^2^, the following settings were used: resolution, 175,000; AGC target, 1E5; maximum injection time, 50 ms; loop count, 15; isolation window, 4.0 *m*/*z*; NCE, 30. The following data-dependent settings were used: underfill ratio, 1%; apex trigger, off; charge exclusion, unassigned, 1, 5, 5–8, >8; peptide match, preferred; exclude isotypes, on; dynamic exclusion, 20.0 s. The raw files obtained from Xcalibur (Thermo Fisher Scientific) were uploaded into MaxQuant (v.1.5.2.8)^77^ and queried against an in-house database containing proteins from 21 plant species including tobacco sequences. The default parameters were used, except that label-free quantification and intensity-based absolute quantification were activated. The MS proteomics data have been deposited to the ProteomeXchange Consortium via the PRIDE^39^ partner repository with the dataset identifier PXD032330.

### Structural figures

The cryo-EM maps were supersampled in Coot for improved smoothness for some figures. All figures showing structural models were prepared using UCSF Chimera^56^ and UCSF ChimeraX^78^.

### Reporting summary

Further information on research design is available in the Nature Portfolio Reporting Summary linked to this article.

## Supplementary information

Supplementary Information Supplementary Figs. 1–18, Table 1, results and list of Supplementary Tables 2–10 supplied as spreadsheets.

Reporting Summary

Supplementary Data 1 Supplementary Tables 2–10.

## Data Availability

The cryo-EM maps for the 40S and 60S subunits and the 80S ribosome with bound tRNAs have been deposited in the Electron Microscopy Data Bank with accession codes EMDB-15674, EMDB-15773 and EMDB-15806, respectively. The atomic models for the 60S and 40S subunits and the actively translating 80S ribosome have been deposited in the PDB under accession codes 8auv, 8azw and 8b2l, respectively. The MS proteomics data have been deposited to the ProteomeXchange Consortium via the PRIDE partner repository with the dataset identifier PXD032330. Dataset ‘N. tabacum BX Sierro 2014 BLAST’ from the SGN database (https://solgenomics.net) was used to obtain RNA sequences for model building. To obtain the protein sequences, the following databases were used: NCBI, Phytozome (https://phytozome.jgi.doe.gov), FernBase (https://www.fernbase.org) and ConGenIE (http://congenie.org). The starting atomic coordinates used to build the tobacco 80S ribosome model were 5m1j, 6ek0 and 5aj0 (PDB). The ribosomal models used for comparative analyses during the study were 6y57, 6qnr, 4v88, 7qiz, 4v6w, 4v8p, 4bts and 4v9d (PDB).

## Code availability

The Python script used for conversion has been uploaded to GitHub (https://github.com/spahnlab/publications).
