# Supplementary material for: Structure of the actively translating plant 80S ribosome at 2.2 Å resolution
Source: Nat Plants. 2023 May 8;9(6):987–1000. doi: 10.1038/s41477-023-01407-y (PMC10281867; doi:10.1038/s41477-023-01407-y)
Supplement: Supplementary file 1 — Supplementary Figs. 1–18, Table 1, results and list of Supplementary Tables 2–10 supplied as spreadsheets. [file 41477_2023_1407_MOESM1_ESM.pdf]

nature plants

Resource https://doi.org/10.1038/s41477-023-01407-y

Structure of the actively translating plant 80S ribosome at 2.2 Å resolution

In the format provided by the authors and unedited

Supplementary Information:

Supplementary Figures 1-18

Supplementary Table 1

List of Supplementary Tables 2-10 supplied as spreadsheets

Extended Data Text

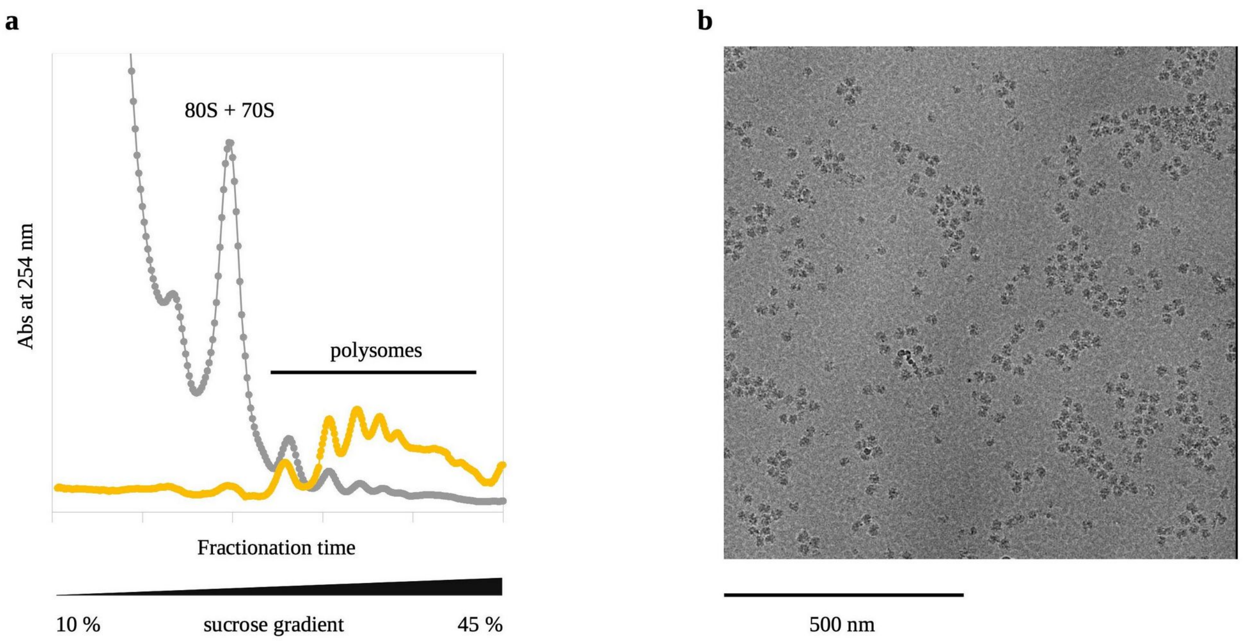

Supplementary Fig. 1.

Total plant polysome sample isolated from the plant cell.

(**a**) Classical sucrose density-gradient analysis of the polysomal sample before (gray line) and after (orange line) short sucrose cushion centrifugation. The sample is enriched in high-molecular-weight components like polysomes after the cushion centrifugation. Fractions corresponding to polysomes were used for the cryo-EM analysis. (**b**) Analytical transmission cryo-EM of plant polysomes after sucrose cushion centrifugation in amorphous ice (120 kV Tecnai Spirit, magnification 42,000x). The micrograph shows distinct clusters of ribosomes, presumably indicative of polysomal chains. A typical micrograph of more than one hundred is shown.

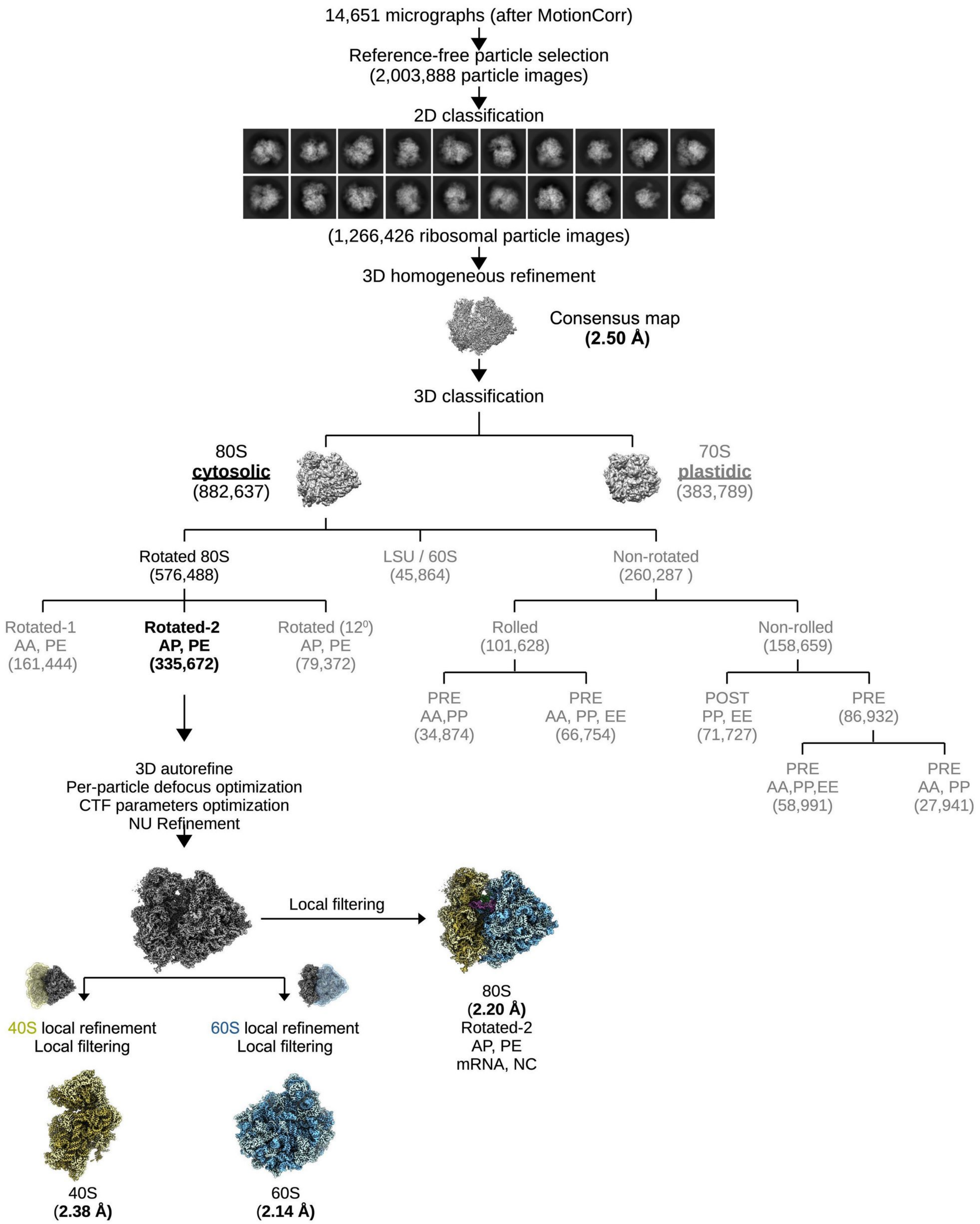

Supplementary Fig. 2.

Cryo-EM data processing workflow.

Particle images of plant polysomes were analyzed by 2D classification in cryoSPARC (for exemplification, 20 classes out of 300 are shown) and sorted into several subclasses using a 3D multi-particle alignment procedure in SPIDER. Several classes representing distinct conformations of elongating 80S ribosome could be identified. The largest homogeneous class representing the 80S ribosome in a rotated-2 conformation with two bound tRNAs was used for refinement in RELION, where per-particle defocus was determined. Subsequently, a non-uniform refinement with optimization of the CTF parameters in cryoSPARC improved the final resolution of the 80S ribosome in rotated-2 conformation. Further local refinement on the 60S (blue) and 40S (yellow) subunits improved map quality and resolution. Masks used for local refinement are indicated as colored mesh superimposed on the ribosome (grey). The number of particles at each stage is shown in brackets. The respective resolution, as defined by gold standard half-map FSC (cutoff of 0.143), is shown for the three final maps. All maps were filtered to the local resolution before further interpretation.

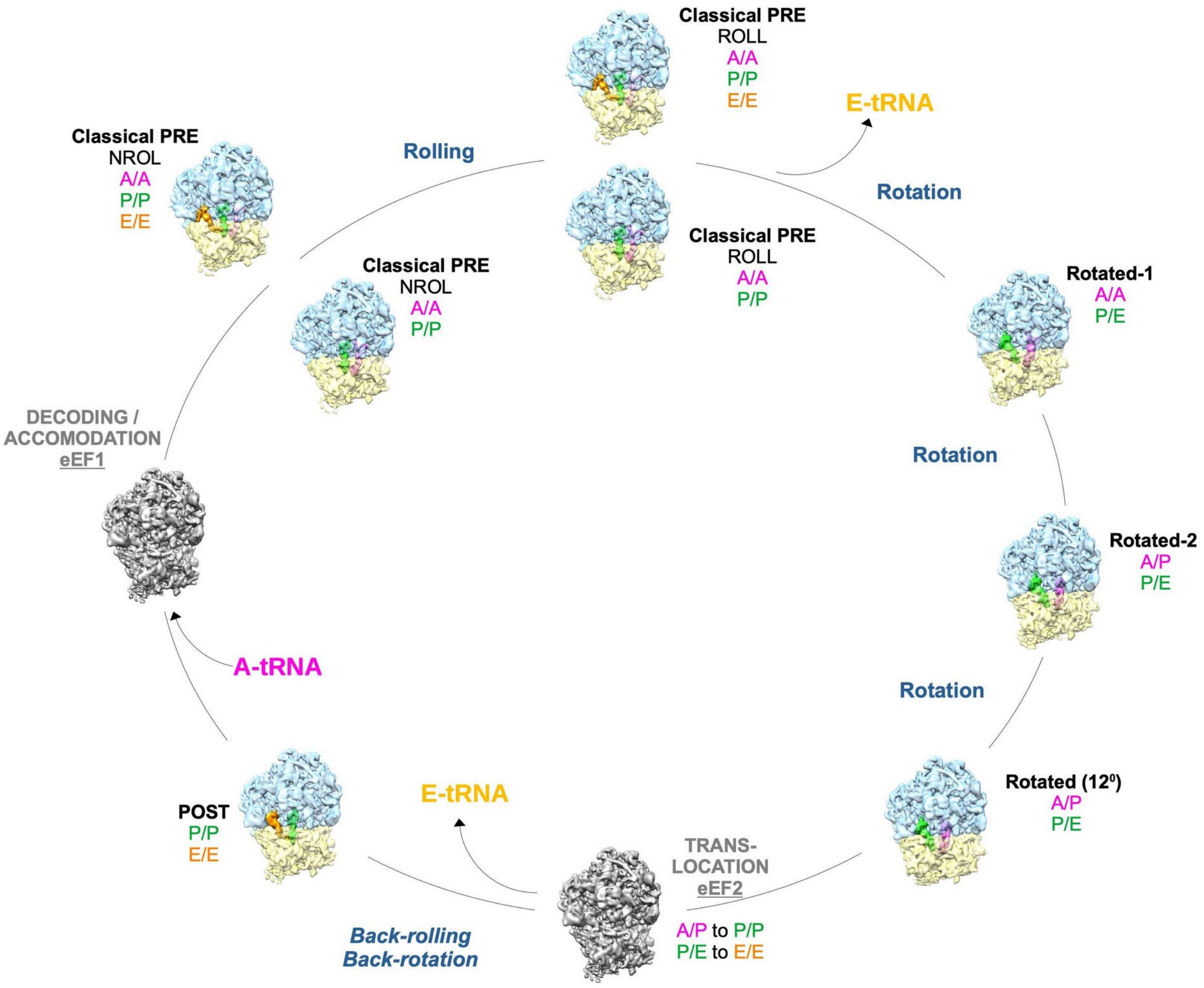

Supplementary Fig. 3.

The experimentally observed elongation cycle from tobacco cytosol.

Overview of the cryo-EM maps in the framework of the elongation cycle. Translocation and decoding-sample-recognition complexes were not observed experimentally and are shown schematically in gray. All maps shown were reconstructed at the pixel size 2.12 Å/px and have a resolution of 5 Å on average. Blue: 60S yellow: 40S, pink: A-site tRNA, green: P-site tRNA, orange: E-site tRNA. mRNA and NC are present in each reconstruction, but not shown here for visual clarity.

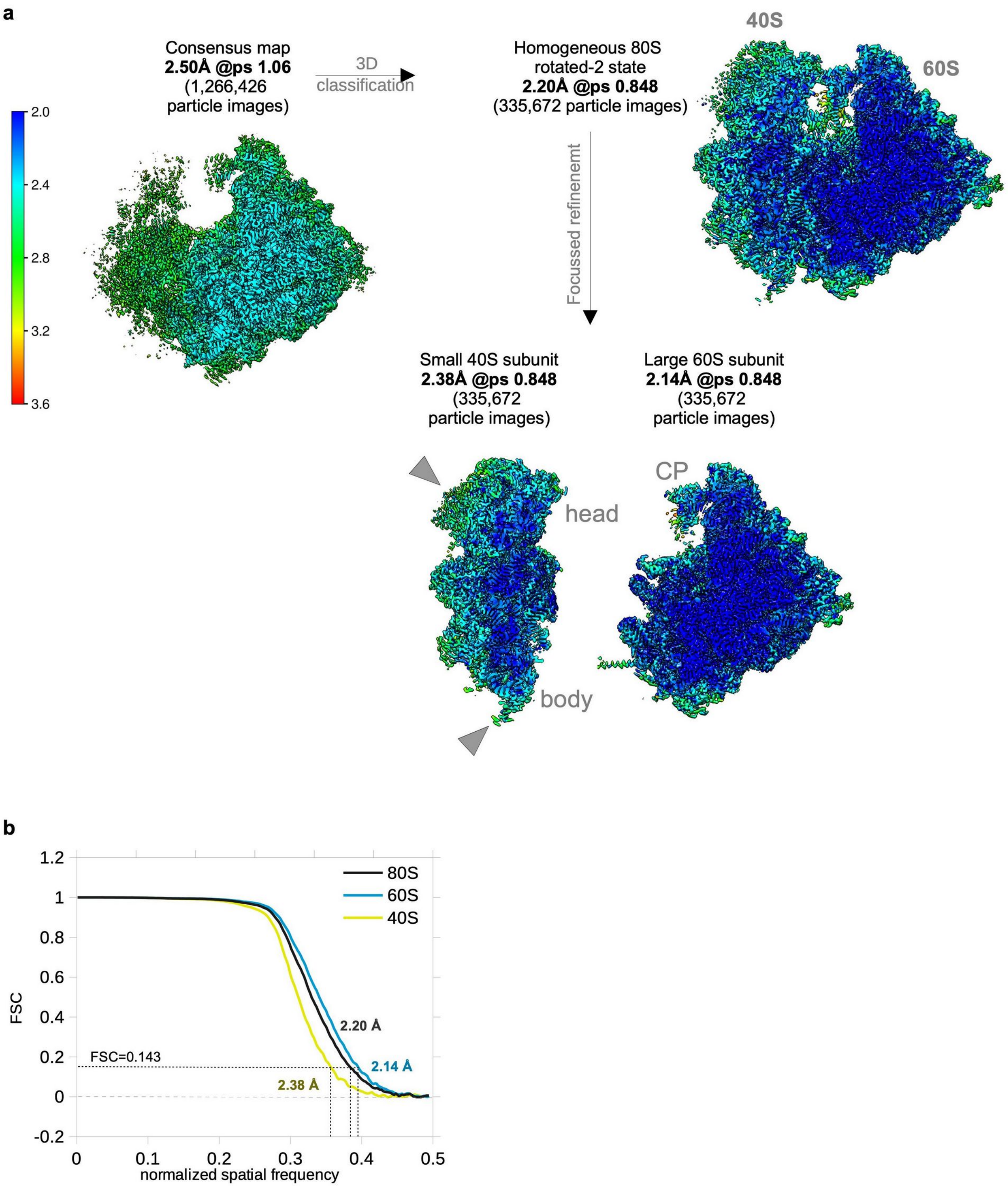

Supplementary Fig. 4.

Resolution of maps.

(**a**) Cutaway view through the cryo-EM density maps of the consensus reconstruction (before the 3D sorting), of the rotated-2 tobacco 80S ribosome and of the 40S (left) and 60S (right) subunits

after separate local refinements in cryoSPARC. The maps are colored by the local resolution as calculated in cryoSPARC. Head and body of the 40S as well as 60S central protuberance (CP) are labeled. Large improvement of the local resolution and overall map quality is achieved by *in silico* sorting and subsequent isolation of highly homogeneous single species, namely the rotated-2 state. Improvement of the 40S head and body distal regions are marked by gray triangles. **(b)** Global resolution for masked maps of the 40S (yellow) and 60S (blue) subunits as well as the rotated-2 80S ribosome (black) as shown by half-map FSC curves. The resolution values are shown for cutoff value of 0.143.

```
a
5`-end of the 5S rRNA gene:
N.tabacum_NCBI            ---------------------------------------TTGGATGCGATCATACCAGCA 21
A.thaliana_NCBI           CTTAAAAAAAGAATATATACGATTGCATTGCATATACTAACGGATGCGATCATACCAGCA 60
N.tabacum_SolGenome       ----------------------------------------CGGATGCGATCATACCAGCA na
N.tabacum_modeled         -----------------------------------------GGATGCGATCATACCAGCA 19

3`-end of the 5S rRNA gene:
N.tabacum_NCBI            AGGATGGGTGACCCCCTGGGAAGTCCTCGTGTTGC------------------------- 116
A.thaliana_NCBI           AGGATGGGTGACCTCCCGGGAAGTCCTCGTGTTGCATCCCTCTTTTTTTTTTTTTTTTTT 180
N.tabacum_SolGenome       AGGATGGGTGACCCCCTGGGAAGTCCTCGTGTTGCATCCCTCTTTTT------------- na
N.tabacum_modeled         AGGATGGGTGACCCCCTGGGAAGTCCTCGTGTTGCATCCC-------------------- 119

b
5`-end of the 5.8S rRNA gene:
N.tabacum_NCBI            CTCGCGCCCC---GTTCGCGGTGCGCGCGGGGGACTTGTGCTTCTTTTGAAACACAAACG 311
A.thaliana_NCBI           TTCGCCTCCCCGGAGACGGAGTGTGGGCGGATGCTGTGCTGCGAACTGAAGTCTAAAACG 275
N.tabacum_SolGenome       CTCGCGCCCC---GTTCGCGGTGCGCGCGGGGGACTTGTGCTTCTTTTGAAACACAAACG na
N.tabacum_modeled         -------------------------------------------------------AAACG 5

3`-end of the 5.8S rRNA gene:
N.tabacum_NCBI            GCCATTAGGCCGAGGGCACGTCTGCCTGGGCGTCACGCATCGCGTCGCCCCCCGCACTCC 491
A.thaliana_NCBI           GCCTTCTGGCCGAGGGCACGTCTGCCTGGGTGTCACAAATCGTCGTCCCTCACCATCCTT 455
N.tabacum_SolGenome       GCCATTAGGCCGAGGGCACGTCTGCCTGGGCGTCACGCATCGCGTCGCCCCCCGCACTCC na
N.tabacum_modeled         GCCATTAGGCCGAGGGCACGTCTGCCTGGGCGTCACGC---------------------- 163

c
5`-end of the 25S rRNA gene:
N.tabacum_NCBI            -------------------------------TACCCGCTGAGTTTAAGCATATCAATAAG 29
A.thaliana_NCBI           GTCCATATAGCGACCCCAGGTCAGGCGGGATTACCCGCTGAGTTTAAGCATATCAATAAG 898
N.tabacum_SolGenome       GCTCCGACCGCGACCCCAGGTCAGGCGGGACTACCCGCTGAGTTTAAGCATATCAATAAG na
N.tabacum_modeled         ---------GCGACCCCAGGTCAGGCGGGACTACCCGCTGAGTTTAAGCATATCAATAAG 51

3`-end of the 25S rRNA gene:
N.tabacum_NCBI            CCTTGCTGSCACGATCCACTG--------------------------------------- 3362
A.thaliana_NCBI           CCTTGCTGCCACGATCCACTGAGATTCAGCCCTTTGTCGCTAAGATTCGACCCTCCCCTA 4270
N.tabacum_SolGenome       CCTTGCTGCCACGATCCACTGAGATTCAGCCCTTTGTCGCTCCGATTCGTCCCTCCCCCT na
N.tabacum_modeled         CCTTGCTGCCACGATCCACTGAGATTCAGCCCTTTGTCGCTCCGATTCGT---------- 3390

d
5`-end of the 18S rRNA gene:
N.tabacum_NCBI            -----------------------------GTCATATGCTTGTCTCAAAGATTAAGCCATG 31
A.thaliana_NCBI           TGAATGCTACCTGGTTGATCCTGCCAGTAGTCATATGCTTGTCTCAAAGATTAAGCCATG 140
N.tabacum_SolGenome       GGAATGCTACCTGGTTGATCCTGCCAGTAGTCATATGCTTGTCTCAAAGATTAAGCCATG na
N.tabacum_modeled         -------TACCTGGTTGATCCTGCCAGTAGTCATATGCTTGTCTCAAAGATTAAGCCATG 53

3`-end of the 18S rRNA gene:
N.tabacum_NCBI            AGGAGAAGTCG------------------------------------------------- 1746
A.thaliana_NCBI           AGGAGAAGTCGTAACAAGGTTTCCGTAGGTGAACCTGCGGAAGGATCATTGTCGATACCT 1900
N.tabacum_SolGenome       AGGAGAAGTCGTAACAAGGTTTCCGTAGGTGAACCTGCGGAAGGATCATTGTCGAAACCT 2399
N.tabacum_modeled         NGGAGAAGTCGTANCAAGGTTTCNGTAGGNGNNCCTGCGGAAGGATCATTG--------- 1808
```

Supplementary Fig. 5.

3`- and 5`- ends of the rRNAs from *N. tabacum*.

Sequences of 5S (**a**), 5.8S (**b**), 25S (**c**), and 18S (**d**) rRNAs of *N. tabacum* and *A. thaliana* from the NCBI database as well as of *N. tabacum* from the SolGenomics database used for modeling and the modeled rRNAs have been aligned. The herein structurally determined 3`- and 5`- ends of the rRNAs from *N. tabacum* are underlined and highlighted in yellow. The missing sequence information for the rRNAs from tobacco available at the NCBI database is highlighted in purple.

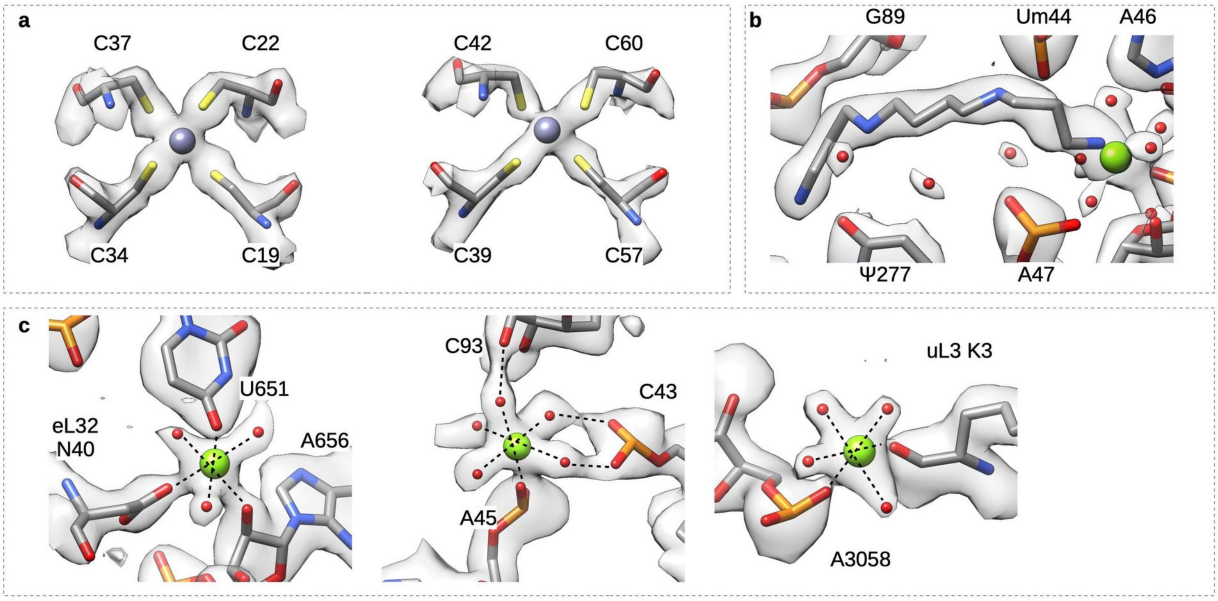

Supplementary Fig. 6

Molecular details assure the quality of the cryo-EM maps and the descendant atomic models.

(a) Zn^{2+} binding pockets of eL37 (left) and eL43 (right) proteins. **(b)** Cryo-EM density of the 60S subunit depicting a density assigned to the polyamine spermine. Examples demonstrate the overall high-resolution of the ligand, nearby solvation and site of Mg^{2+}association. **(c)** Gallery of Mg^{2+} coordination states observed in the 60S subunit. Examples include magnesium ions with different levels of direct coordination to the rRNA or RPs.

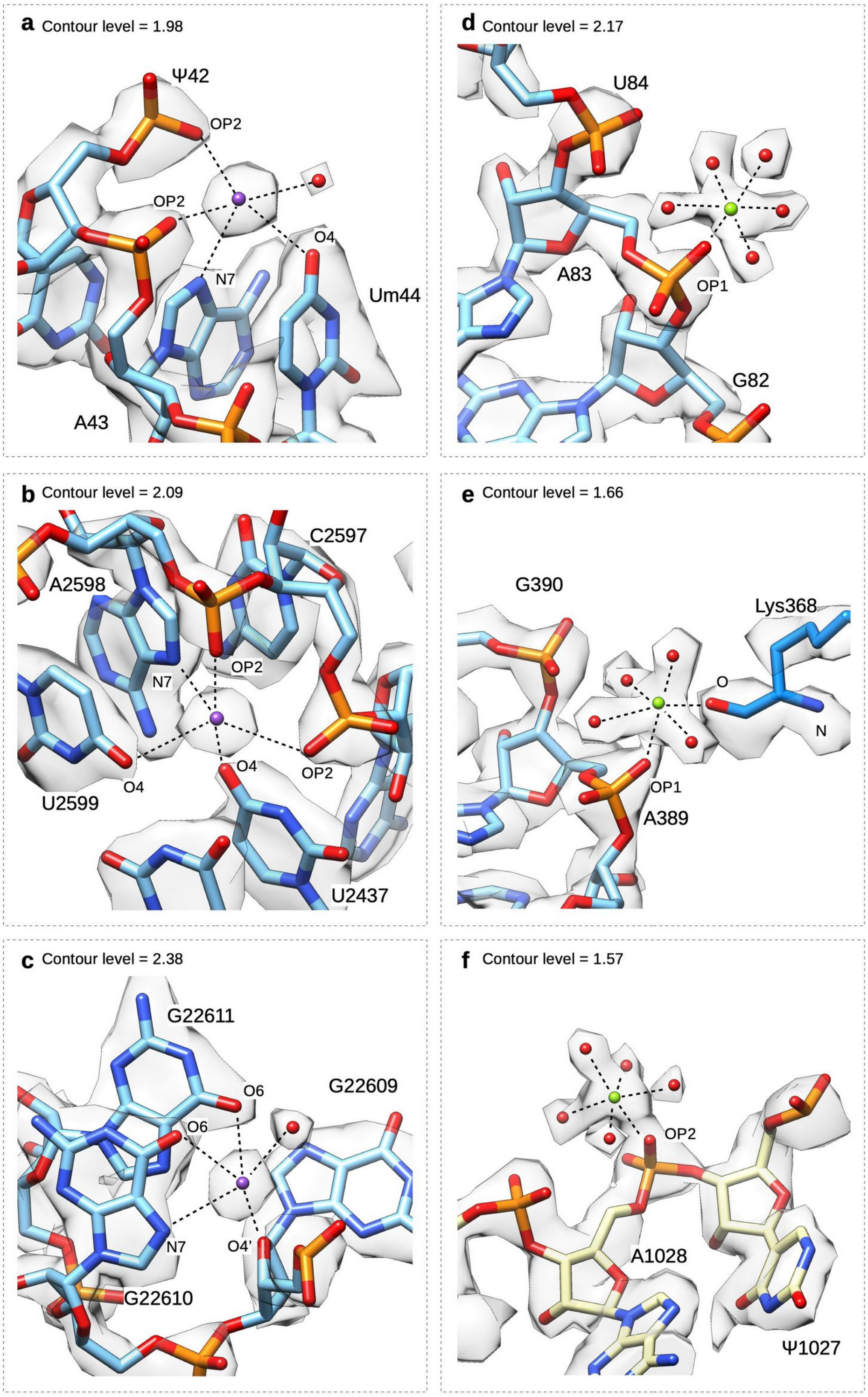

Supplementary Fig. 7

Metal ions bound to the plant 80S ribosome.

Gallery of putative K^{+} **(a-c)** and Mg^{2+} **(d-f)** ions observed in the tobacco 80S ribosome. Examples include magnesium (green spheres) and potassium (purple spheres) ions with different levels of

direct coordination to the rRNA or RPs. Blue: 60S, yellow: 40S, red spheres: water molecules.

Contour level for the cryo-EM density is indicated separately for each example.

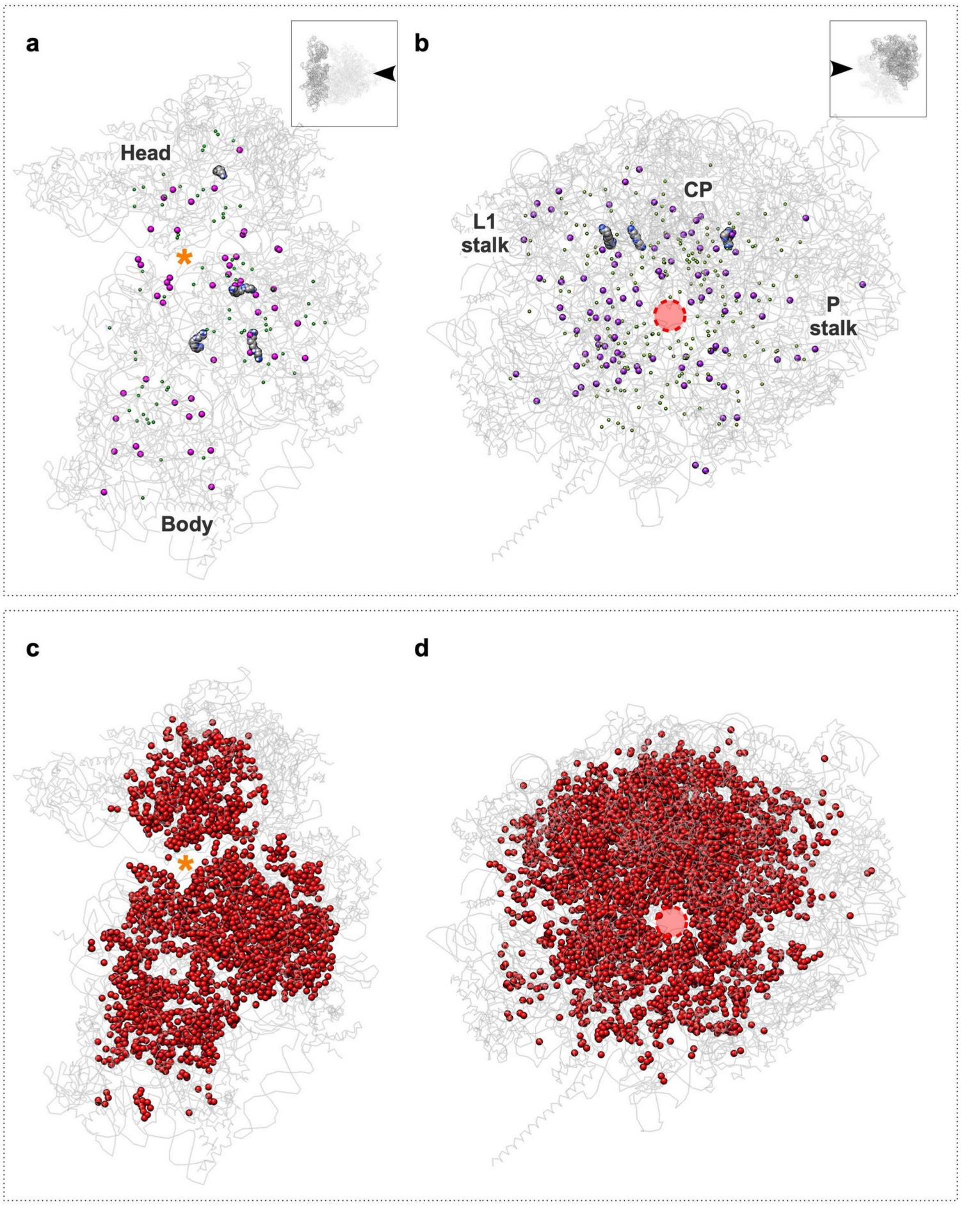

Supplementary Fig. 8.

Solvation of the 80S ribosome.

In **(a)** and **(b)**, the positions of polyamines (gray carbon and blue nitrogen), Mg^{2+} ions (green), and other metal ions (likely K^{+} ions, purple) are shown in the 40S and 60S subunits, respectively. In **(c)** and **(d)**, the solvation of the 40S and 60S subunits with solvent oxygen atoms is shown as red spheres. The mRNA entry site is marked by an orange asterisk and the NC tunnel is marked by a red circle. Structural landmarks are marked (head and body in the 40S subunit, CP, L1- and P-stalks in the 60S subunit).

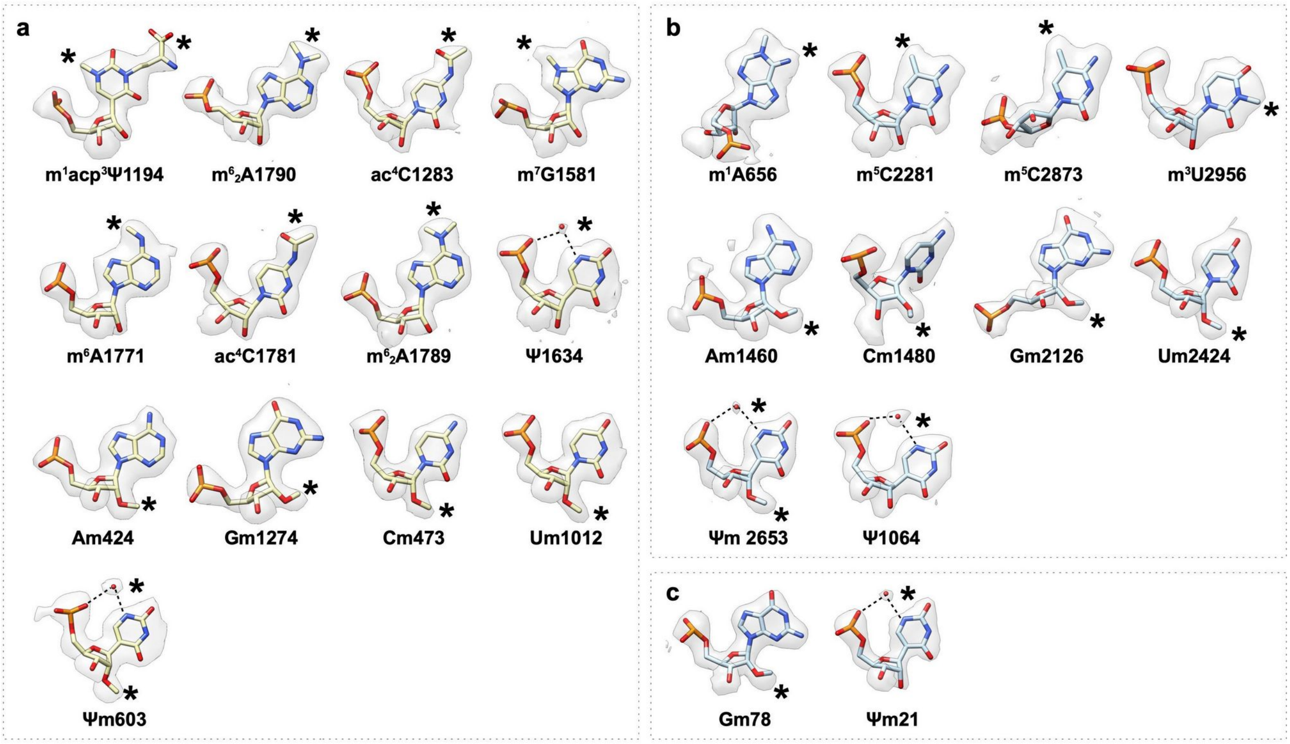

Supplementary Fig. 9.

Gallery of post-transcriptionally modified nucleotides from the 40S and 60S subunits.

Examples of 2′-O-ribose methylation, pseudouridylation, base methylation and acetylation in 18S (**a**), 25S (**b**) and 5.8S (**c**) rRNAs. The additional group in each nucleotide is marked by an asterisk. The characteristic hydrogen bonding in the N1 position in pseudouridines (Ψ) is shown.

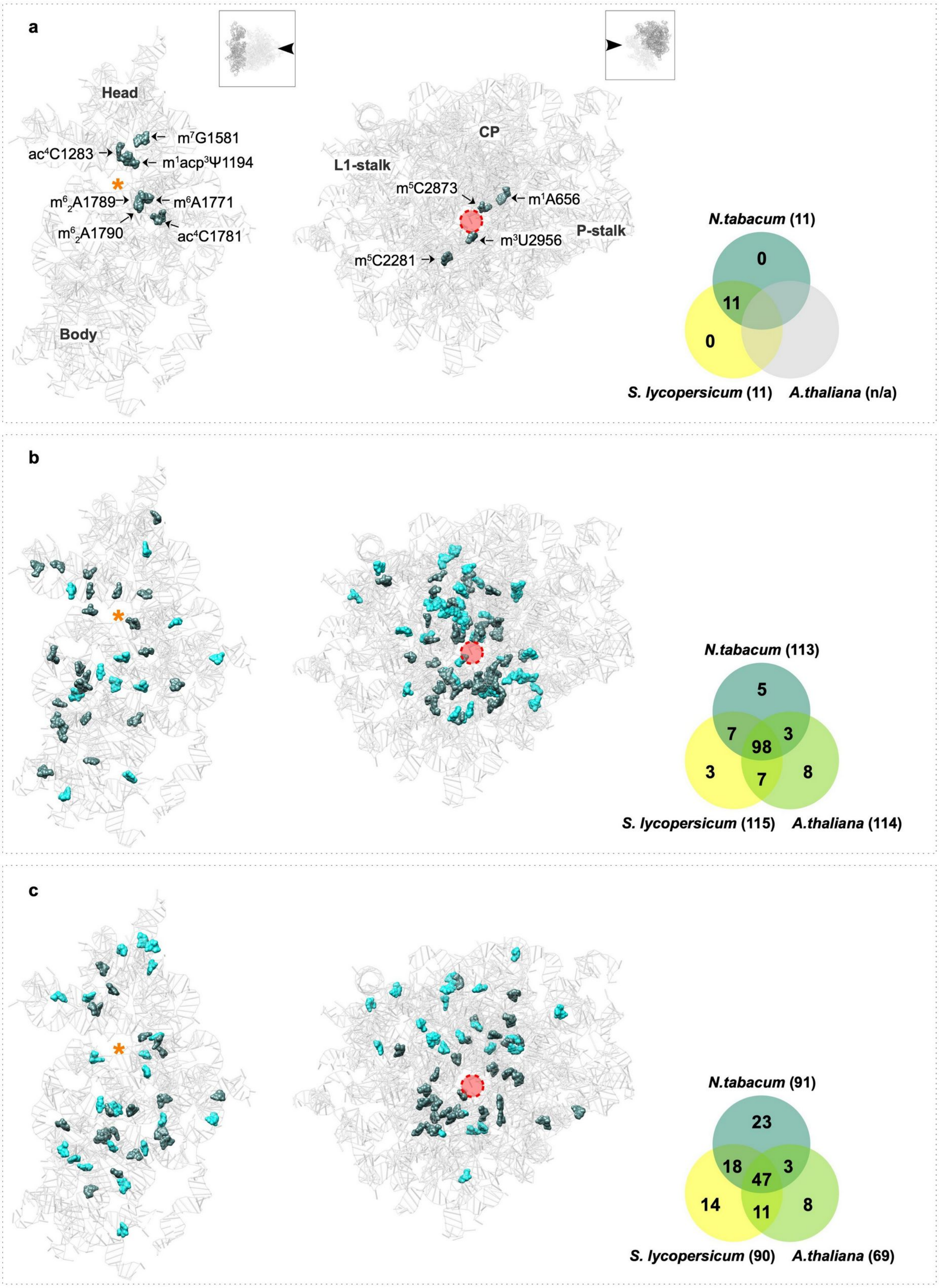

Supplementary Fig. 10.

Chemical modifications of the rRNA in the tobacco 40S and 60S ribosomal subunits.

Positions of base modifications **(a)**, 2′-O-methylation **(b)**, and pseudouridylation **(c)**, found in the rRNAs from tobacco 40S (left) and 60S (right) subunits, respectively. Venn diagrams show conservation of base modifications sites **(a)**, 2′-O-methylation sites **(b)** and of pseudouridylation sites **(c)** between tobacco, tomato, and Arabidopsis. The rRNA components of the 40S and 60S subunits are shown in gray as a ribbon. Modifications conserved between tobacco and yeast, or tobacco and human are shown in dark cyan. Modifications found only in tobacco are shown in bright cyan. The mRNA entry site is marked by an orange asterisk, the NC tunnel is marked by a red circle. Selected structural landmarks are shown (head and body in the 40S subunit, CP, L1- and P-stalks in the 60S subunit). n/a – information not available.

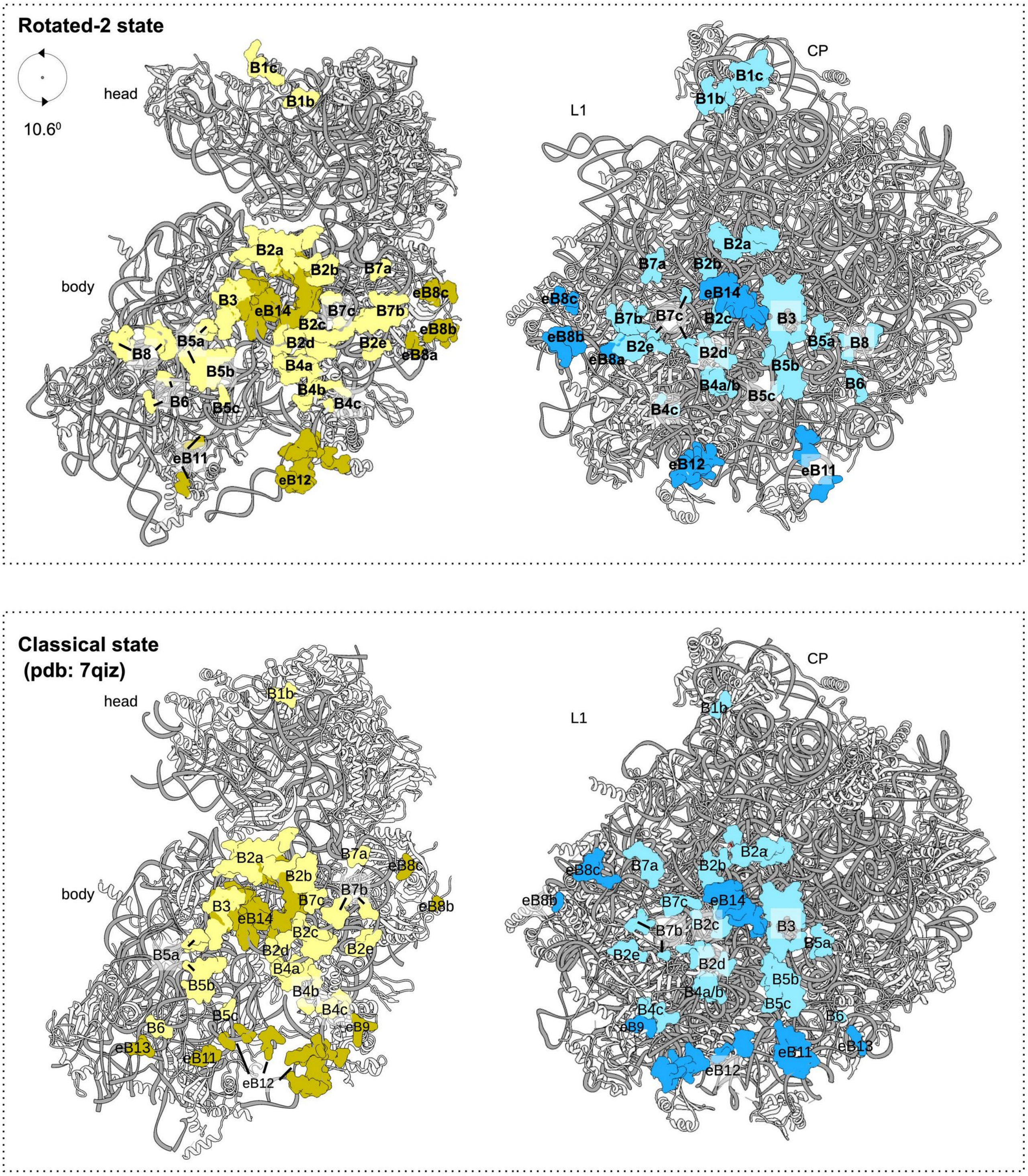

Supplementary Fig. 11.

Intersubunit bridges of the plant 80S ribosome in rotated-2 and classical conformation.

Intersubunit bridges are shown for the rotated-2 80S ribosome from tobacco (top panel) and for the non-rotated 80S ribosome from tomato (pdb: 7qiz; lower panel). Atomic models of the small

subunits (left) and large subunits (right) from tobacco and tomato ribosomes are shown in a ribbon representation; RPs are shown in light gray, rRNA in dark gray. Universally conserved bridges are numbered B1-B8 (light yellow for the small subunit, light blue for the large subunit), and eukaryotic-specific bridges are numbered eB8-eB14 (dark yellow for the small subunit, dark blue for the large subunit). Small subunit body and head domain as well as the large subunit L1 stalk and central protuberance (CP) are labeled. The degree of rotation is indicated.

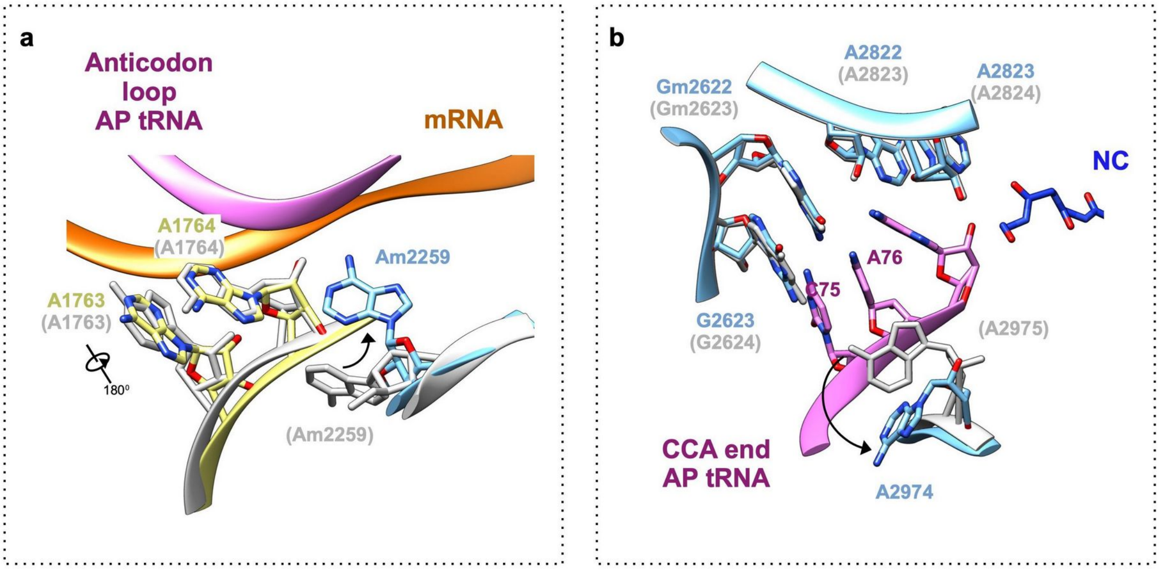

Supplementary Fig. 12

Structural differences in tRNA binding pockets in translating and inactive ribosome.

(a) The binding of tRNA and mRNA to the decoding center of the tobacco ribosome is supported by elements of the small and large subunits. Nucleotides Am2259 from 25S rRNA and A1763 from 18S rRNA change their orientations upon binding to the ligands. **(b)** PTC center of the ribosome with bound tRNA. Nucleotide A2974 adopts an optimal position for binding the CCA-terminus of the tRNA to the P-loop. The elements of the 25S (blue) and the 18S (yellow) rRNAs are shown along with the anticodon loop A/P-site tRNA (pink) and the mRNA (orange). Atomic model of tobacco is shown in color; tomato model (pdb: 7qiz) is underlaid in gray.

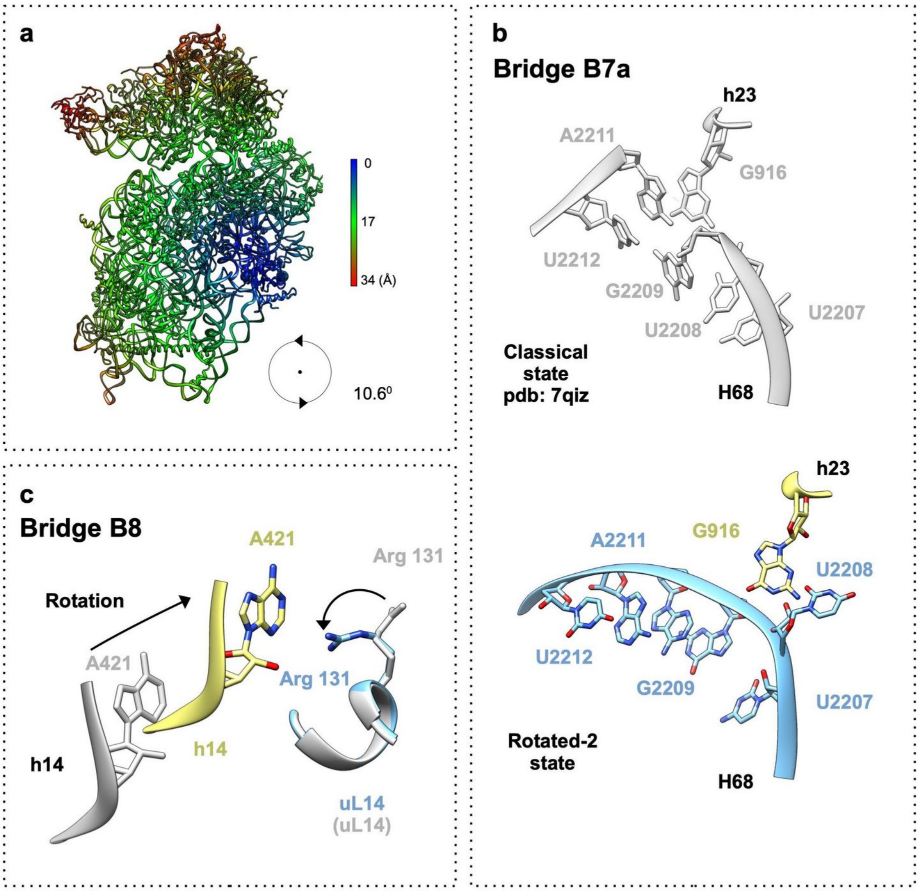

Supplementary Fig. 13

Structural changes in the ribosome associated with intersubunit rotation.

(a) Comparison of the 40S subunit positions in the rotated-2 (tobacco) to the classical (tomato) state represented by ribbons. Comparisons are based on a common 60S alignment. The arrow indicates the direction of movement during rotation. The distance changes in the 40S subunit positions resulting from the rigid body transformation are color-coded in Å units. **(b)** and **(c)** Two examples illustrating structural changes during 40S rotation. **(b)** The bridge B7a is formed by the interaction between G916 of 18S rRNA and A2211 of 25S rRNA in the classical state (upper panel) or U2208 of 25S rRNA in the rotated state (lower panel). Note that U2208 is flipped out to form the interaction. **(c)** The B8 bridge is formed as a result of the interaction between A421 of 18S rRNA and Arg131 of uL14 in the rotated state. The Arg131 side chain rotamer changes upon rotation to facilitate this interaction. Comparisons are based on a common 60S alignment. The elements of the

25S (blue) and the nascent chain (NC, red) are shown. The atomic model of tobacco is shown in color; the tomato model (pdb: 7qiz) is shown in gray.

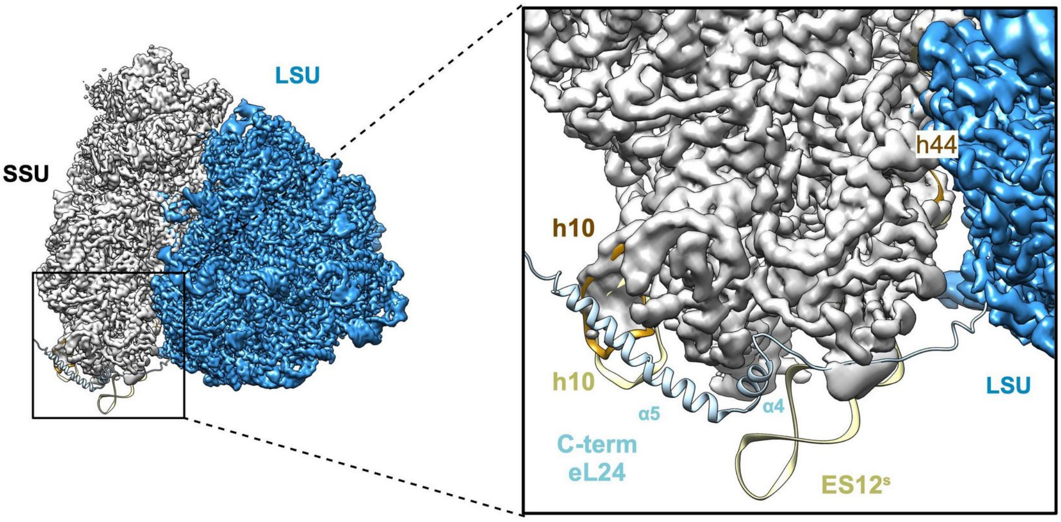

Supplementary Fig. 14.

Cryo-EM density showing absence of the large part of eL24 in the tobacco 80S ribosome.

The cryo-EM density map of the tobacco 80S ribosome (in surface representation) reveals the absence of the C-terminus of the eL24 protein and shows that the density of the expansion segment $ES12^S$ is lowly resolved. SSU, small subunit (gray). LSU, large subunit (blue). Atomic models for helix h10 and $ES12^S$ are shown for tobacco (in brown) and yeast (pdb: 4v88/ribosome B; in yellow). C-terminus of eL24, which extends towards the SSU and wraps around it, is shown for yeast (pdb: 4v88/ribosome B; in light blue). Two α-helices (α4 and α5) responsible for facilitating contacts with the SSU are labeled.

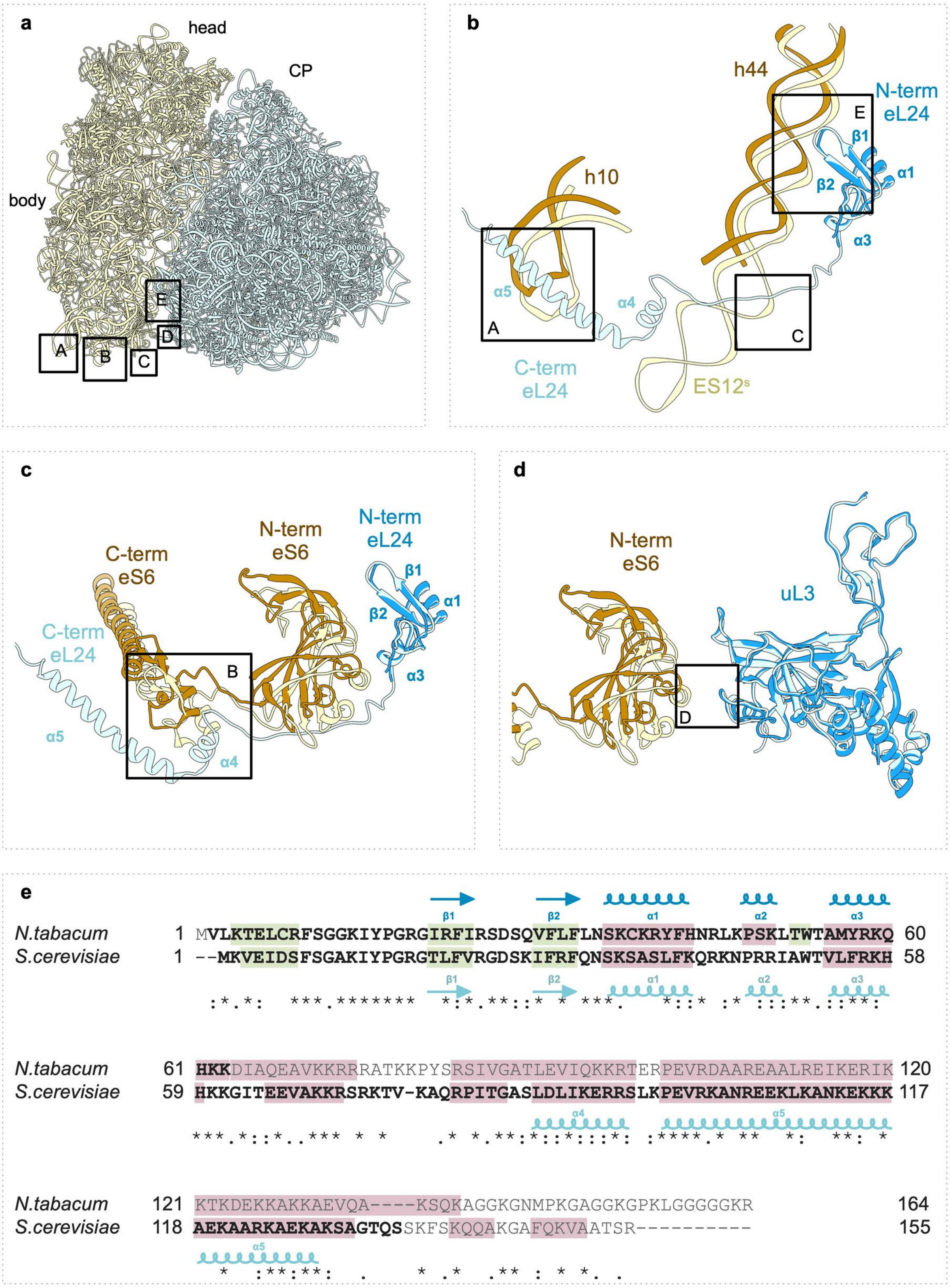

Supplementary Fig. 15.

Comparison of the ribosomal eB13 and B6 bridges between different eukaryotes.

(a) Interaction sites between the 40S (yellow) and the 60S (blue) subunits representing the eukaryote-specific bridge eB13 (rectangular selections A-D) and universal bridge B6 (rectangular selection E) are mapped onto the 80S structure from tobacco. **(b-d)** 80S structures from tobacco (brown for the SSU and dark blue for the LSU) and yeast (pdb: 4v88/ribosome B; yellow for the SSU and light blue for the LSU) are aligned on the 60S subunit. The two bridges eB13 and B6 are formed by several interactions between the eukaryote-specific protein eL24 and universal protein uL3 on the LSU and the eukaryote-specific protein eS6 as well as 18S rRNA helices h44, h10, and $ES12^S$ on the SSU in yeast. In the rotated ribosome from tobacco, the C-terminal helix and flexible linker of eL24 are not visible, resulting in the loss of interactions in rectangular selections A-C. Two other interactions (rectangular selections D and E) are also weakened or missing in tobacco due to a larger degree of rotation of the SSU and displacement of the corresponding components from h44 and the N-terminus of eS6 relative to the LSU. **(e)** Alignment of eL24 from tobacco and yeast shows conservation in both N- and C-terminal parts, as well as in the flexible linker (residues 59–79 in yeast). Structured and unstructured elements are shown in black and gray, respectively. Positions of α-helices and β-sheets are depicted in dark blue for the tobacco 80S structure and light blue for the yeast structure (pdb: 4v88/ribosome B). Prediction for the secondary structure elements are highlighted in green for β-sheets and pink for α-helices.

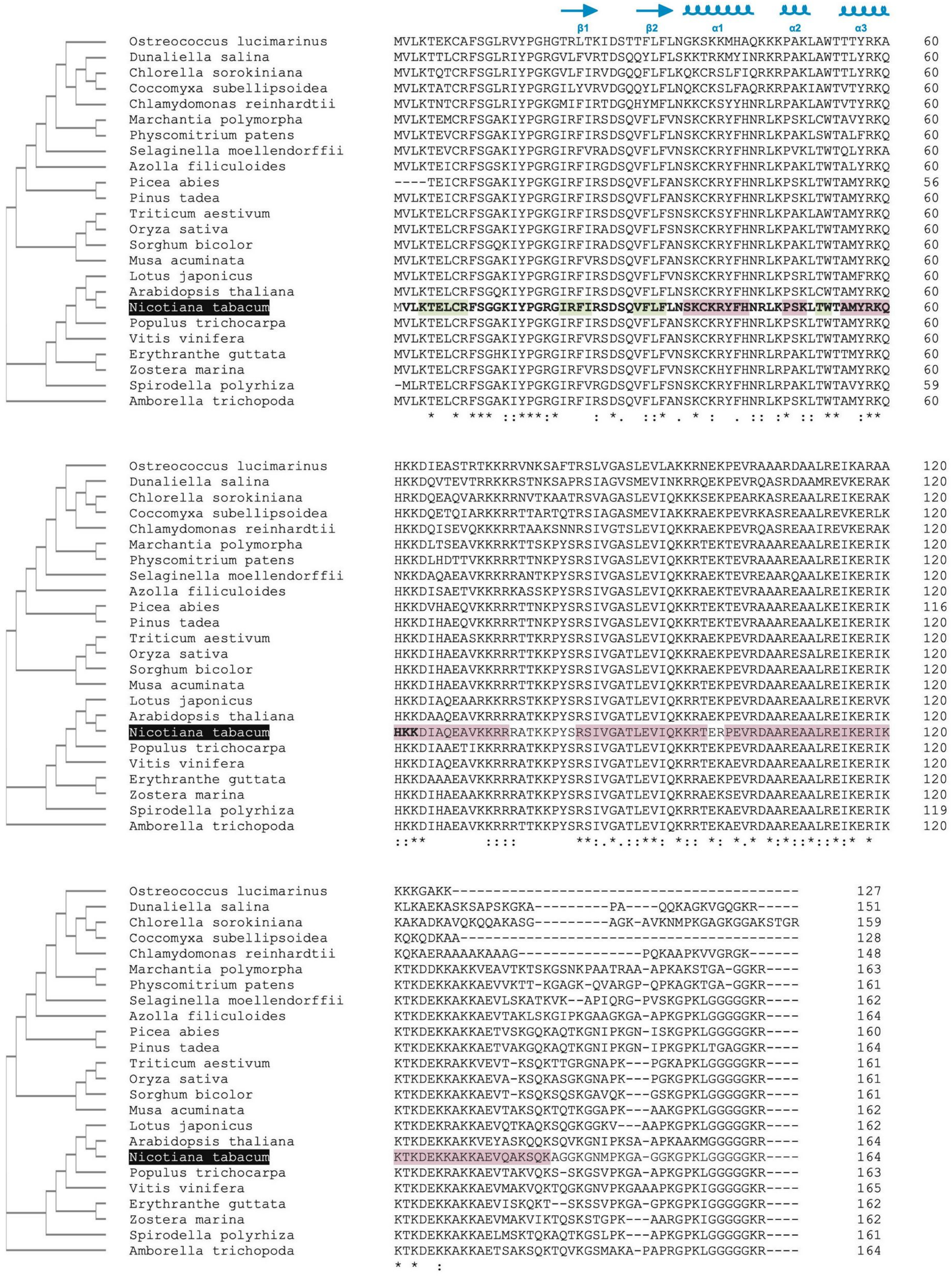

Supplementary Fig. 16.

Conservation of eL24 in vascular plants.

Sequences alignment shows high level of conservation of eL24 among vascular plants. Positions of α-helices and β-sheets are depicted in dark blue for the tobacco 80S structure. Prediction for the secondary structure elements are highlighted in green for β-sheets and pink for α-helices.

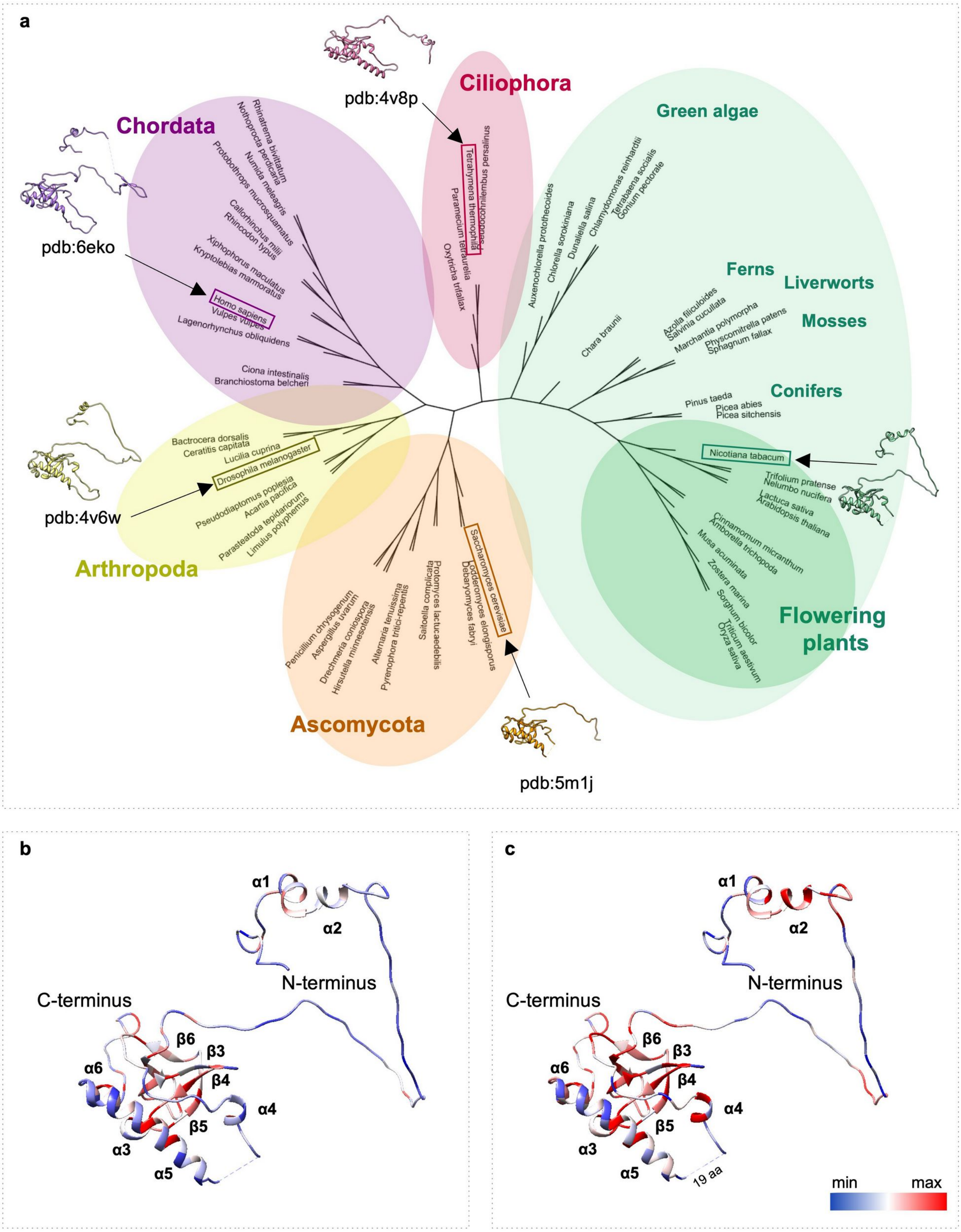

Supplementary Fig. 17.

Sequence conservation of the eukaryote-specific eL6 protein.

(a) Phylogenetic tree based on eL6 amino acid sequences from various species. Pink: phylum Ciliophora (with representative structure from *T. thermophila*) from the protist kingdom. Purple and yellow: phylum Chordata (*H. sapiens*) and phylum Arthropoda (*D. melanogaster*), correspondingly, both from the animal kingdom. Orange: phylum Ascomycota (*S. cerevisiae*) from the fungi kingdom. Dark green: division (phylum) Magnoliophyta (flowering plants; *N. tabacum*) from the plant kingdom. Light green: other divisions of plants. Species for which a structure is available are marked by a rectangle. Corresponding structures are shown. In **(b)** and **(c)** eL6 sequence conservation over all species and in plants is mapped on the protein structure. Conservation level of each amino acid is mapped on the eL6 structure from *N. tabacum*. The color bar shows a gradient from minimal (blue) to maximal (red) conservation level among selected organisms. Selected α-helices and β-sheets are indicated, and C- and N-termini are labeled.

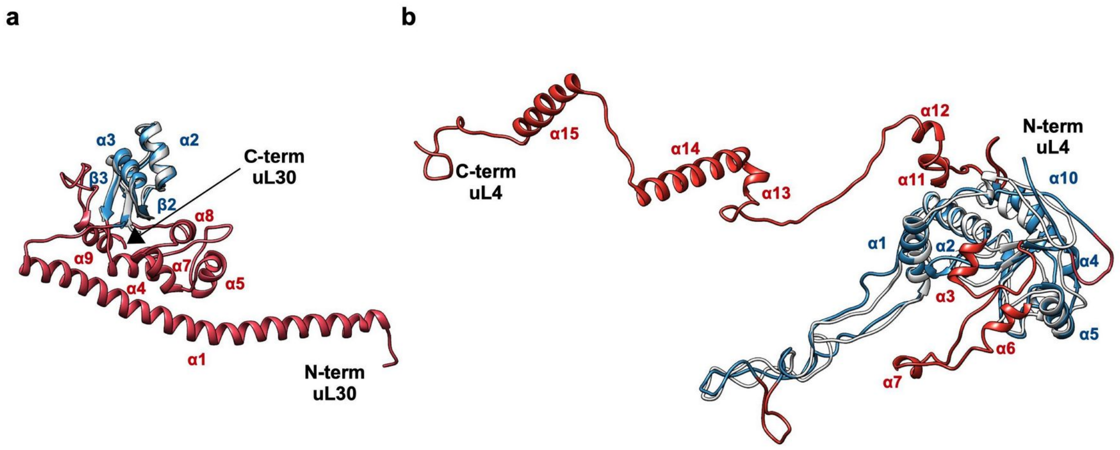

Supplementary Fig. 18.

Eukaryote-specific extensions and insertions in uL4 and uL30 proteins.

In **(a)** and **(b)** homologous *E. coli* protein models (pdb: 4v9d, gray) are overlaid with *N. tabacum* models for proteins uL30 and uL4, respectively. Parts common to prokaryotes and eukaryotes are blue-colored. Eukaryote-specific extensions and insertions are red-colored. C-and N-termini for both proteins are shown. Selected secondary elements (α-helices and β-sheets) are labeled.

Supplementary Table 1.

Data collection, processing and model refinement statistics.

	Small 40S subunit from tobacco (EMDB-15674) (PDB: 8AUV)	Large 60S subunit from tobacco (EMDB-15773) (PDB: 8AZW)	Rotated-2 80S ribosome from tobacco (EMDB-15806) (PDB: 8B2L)
**Data collection and processing**			
Magnification	81000	81000	81000
Voltage (kV)	300	300	300
Electron exposure (e–/Å^2)	27	27	27
Defocus range (μm)	0.5-1.8	0.5-1.8	0.5-1.8
Pixel size (Å)	0.53	0.53	0.53
Symmetry imposed	no	no	no
Initial particle images (no.)	2003888	2003888	2003888
Final particle images (no.)	335291	335291	335291
Map resolution (Å)	2.38	2.14	2.20
FSC threshold	0.143	0.143	0.143
Map resolution range (Å)	2.0 – 3.2	1.8 – 2.8	1.8 – 3.4
**Refinement**			
Initial model used (PDB code)	5m1j (rRNA, r-proteins), 6ek0 (r-proteins)	5m1j (rRNA, r-proteins), 6ek0 (r-proteins)	5m1j (rRNA, r-proteins), 6ek0 (r-proteins), 5aj0 (tRNAs)
Model resolution (Å)	2.6	2.27	2.30
FSC threshold	0.5	0.5	0.5
Model resolution range (Å)	n/a	n/a	n/a
Map sharpening *B* factor (Å^2)	74.7	61.2	61.9
Model composition			
Non-hydrogen atoms	75,611	131,052	209,939
Protein residues	4,804	6,420	11,268
Nucleotides	1,640	3,482	5,279
Ligands			
Zn:	3	4	7
SPM:	3	2	5
K:	47	94	141
SPD:	1	1	2
UNK (nascent chain):	-	24	24
Mg:	76	59	135
B factors (Å^2)			
Protein	6.31	10.52	34.06
Nucleotide	4.69	16.83	40.27
Ligand	5.83	15.06	24.19
R.m.s. deviations			
Bond lengths (Å)	0.002 (1)	0.002 (4)	0.002 (0)
Bond angles (°)	0.526 (11)	0.486 (5)	0.492 (12)
Validation			
MolProbity score	1.16	1.13	1.16
Clashscore	3.74	3.42	3.73
Poor rotamers (%)	0.22	0.18	0.38
Ramachandran plot			
Favored (%)	98.43	98.61	98.26
Allowed (%)	1.55	1.37	1.72
Disallowed (%)	0.02	0.02	0.02

Supplementary Table 2.

rRNA and tRNA sequences from *N. tabacum* used for the model building.

Supplementary Table 3.

Proteins from the *N. tabacum* ribosomal small 40S subunit, used for model building.

Supplementary Table 4.

Proteins from the *N. tabacum* ribosomal large subunit, used for the model building.

Supplementary Table 5.

Overview of molecular interactions of the plant 80S ribosome with A/P and P/E tRNAs.

Supplementary Table 6.

Positions and type of nucleotides modifications identified in the tobacco 80S ribosome structure in comparison to Arabidopsis data.

Supplementary Table 7.

Conservation of modified nucleotides in rRNAs among different eukaryotes: tobacco, human, and yeast.

Supplementary Table 8.

Comparison of molecular details of the plant 80S ribosome in the rotated-2 (tobacco) and classical (tomato) conformation.

Supplementary Table 9.

Compositional overview of 80S ribosomes from different eukaryotes.

Supplementary Table 10.

List of sequences used in this study.

Extended Data:

Intersubunit bridges in the rotated configuration of the plant 80S ribosome

Bridge eB8 is localized at the far end of the SSU body platform (Extended data Fig. 1). In yeast, this bridge is formed by contacts between protein eS1 and two components of the LSU: $ES31^L$ and eL43[12]. In tobacco, bridge eB8 is additionally sustained by the interaction of eS1 with the C-terminus of eukaryote-specific protein eL8. Interestingly, both tobacco and human eL8 (pdb: 6y57[16]) have a longer C-terminus compared to yeast, resulting in an extra component of eB8 in these structures.

Below the mRNA exit site, one of the solvent-exposed bridges eB12 is formed by multiple interactions between eL19, eS7 and $ES6^S$ (Extended Data Fig. 2). Compared to yeast[12], the distal part of the tobacco C-terminal helix of eL19 is displaced by approximately 6 Å (distance is measured between the two respective Glu179). This shift reflects a different rotation degree of the SSU in the two structures and supports the flexible nature of the lateral bridges. However, the interaction interface remains the same, and multiple charged arginines and lysines of eL19 coordinate the hydrogen bonds with various elements from $ES6^S$ (Extended Data Fig. 2b). These include, for example, a contact between Arg163 and U817, as shown for the mammalian 80S ribosome in post-translocational state[22].

Bridge eB11 is located at the bottom of the ribosome, near the footing of flexible $ES27^L$ (Extended Data Fig. 3). In the rotated 80S ribosome from yeast[12] it is mainly formed by interactions between protein eS8 and $ES41^L$. Differing from this, components of the tobacco bridge eB11 appear to have weaker interactions due to a larger distance between the corresponding components. Moreover, also in tobacco 80S, H63 forms an interaction with eS8 (Extended Data Fig. 3b), resembling the situation in yeast 80S, which is less rotated (pdb: 4v88[12], ribosome A, degree of rotation 5.3°).

Architecture of plant-specific expansion segments and associated proteins

The N-terminus of uL4 represents a conserved globular core with a large loop (in tobacco: amino acids 49-119, between the α1- and α2-helices), protruding through the ribosome towards the NC exit tunnel (Supplementary Fig. 17b, Extended Data Fig. 4). In eukaryotes, this loop has an insertion, which narrows the NC tunnel. Moreover, eukaryote-specific C-terminal extensions of various lengths are present in 5 structures of 80S ribosomes (Extended Data Fig. 4). In tobacco, this extension is well structured and tightly bound to $ES7^L$: the α14-helix of uL4 passes between the $ES7^L$c-e and $ES7^L$b branches, supporting their interaction, while α15-helix of uL4 is bound to $ES7^L$b and almost reaches protein uL16 with its most distal C-terminal end. The only interaction between those two could be formed between Asn396 from uL4 and Arg169 from uL16 (3.84 Å distance) (Extended Data Fig. 4b). Similar to Tetrahymena, the tobacco uL4 C-terminal extension forms two long helices, both of which interact with $ES7^L$b, but in contrast, its most distal C-terminal end binds tightly to the uL16 protein (Extended Data Fig. 4f).

In uL4 proteins from human and Drosophila, the part corresponding to the α14-helix in tobacco has similar fold and interactions, whereas the part corresponding to the α15-helix in tobacco is either unstructured or does not exhibit interaction with $ES7^L$b (Extended Data Fig. 4c, d). In yeast, both helices are much shorter, though the C-terminal end interacts with the respective $ES7^L$b (Extended Data Fig. 4e). The sequence alignment of uL4 from 5 different eukaryotes also reveals a high level of diversity in its C-terminal part (Extended Data Fig. 4g).

The subsequent sequence conservation analysis that included more species revealed that uL4 strongly differs in sequence and length among the analyzed organisms, with the phylogenetic analysis clearly separating the analyzed species into distinct phyla (Extended Data Fig. 5a). The N-terminal domain of uL4, including the loop reaching the NC tunnel, has a remarkable conservation level among all eukaryotes (Extended Data Figs. 4g, 5b). In contrast, the C-terminal eukaryote-specific extension of uL4 seems to be not conserved among eukaryotes. However, among the

members of the plant kingdom, this extension displays a higher level of conservation, especially in its most distal part (Extended Data Fig. 5b-c). Based on this data, it is likely that the structure and specific interaction between the eukaryote-specific extension of uL4 and ribosomal elements, including branches of $ES7^L$, are preserved in all species of the green lineage. Interestingly, despite a very low level of sequence conservation in the eukaryote-specific extension of the uL4 protein among different kingdoms, it forms remarkably similar α-helical structure elements, interacting with $ES7^L$ branches and stabilizing them.

Protein uL30 has increased four times in size through evolving eukaryote-specific C- and N-terminal extensions. Its eukaryotic C-terminal end is composed mostly of α-helices (in tobacco: α4–α9; Supplementary Fig. 17a) and forms a globular structure (Extended Data Fig. 6b). This part adds up to the conserved core and thus reaches the $ES7^L$b branch, stabilizing it on the ribosomal surface. The eukaryotic N-terminal end of uL30 forms a very long α1-helix, which in tobacco reaches the distal part of $ES7^L$a and therefore paves through all three branches of $ES7^L$ (a, b, and c–e). Interestingly, only in the tobacco structure, the N-terminal end of uL4 directly binds to the eL28 protein (Extended Data Fig. 6b). eL28 stabilizes the $ES7^L$a branch, and its absence in *S. cerevisiae* results in an unstructured ES7La helix in the corresponding 80S structure (Extended Data Fig. 6e). In the other four reported structures, interactions of uL30 with eL28 and $ES7^L$a were not observed. However, the uL30 proteins exhibit remarkable overall structural similarity among different eukaryotes. Interestingly, despite the high level of structural similarity for both N- and C-terminal eukaryote-specific extensions, only the C-terminus reveals a high level of sequence conservation too, while the N-terminus sequence seems to be less conserved when either eukaryotes of all kingdoms or only plants are analyzed (Extended Data Fig. 7).
