# Supplementary material for: Structure of the actively translating plant 80S ribosome at 2.2 Å resolution
Source: Nat Plants. 2023 May 8;9(6):987–1000. doi: 10.1038/s41477-023-01407-y (PMC10281867; doi:10.1038/s41477-023-01407-y)
Supplement: Supplementary file 2 — Reporting Summary [file 41477_2023_1407_MOESM2_ESM.pdf]

nature portfolio

Corresponding author(s): Julia Smirnova, Ralph Bock, Christian TM Spahn, Reimo Zoschke

Last updated by author(s): 22.03.2023

Reporting Summary

Nature Portfolio wishes to improve the reproducibility of the work that we publish. This form provides structure for consistency and transparency in reporting. For further information on Nature Portfolio policies, see our Editorial Policies and the Editorial Policy Checklist.

Statistics

For all statistical analyses, confirm that the following items are present in the figure legend, table legend, main text, or Methods section.

n/a	Confirmed	
☒	☐	The exact sample size (*n*) for each experimental group/condition, given as a discrete number and unit of measurement
☒	☐	A statement on whether measurements were taken from distinct samples or whether the same sample was measured repeatedly
☒	☐	The statistical test(s) used AND whether they are one- or two-sided<br>*Only common tests should be described solely by name; describe more complex techniques in the Methods section.*
☒	☐	A description of all covariates tested
☒	☐	A description of any assumptions or corrections, such as tests of normality and adjustment for multiple comparisons
☒	☐	A full description of the statistical parameters including central tendency (e.g. means) or other basic estimates (e.g. regression coefficient) AND variation (e.g. standard deviation) or associated estimates of uncertainty (e.g. confidence intervals)
☒	☐	For null hypothesis testing, the test statistic (e.g. *F*, *t*, *r*) with confidence intervals, effect sizes, degrees of freedom and *P* value noted<br>*Give P values as exact values whenever suitable.*
☒	☐	For Bayesian analysis, information on the choice of priors and Markov chain Monte Carlo settings
☒	☐	For hierarchical and complex designs, identification of the appropriate level for tests and full reporting of outcomes
☒	☐	Estimates of effect sizes (e.g. Cohen's *d*, Pearson's *r*), indicating how they were calculated

Our web collection on statistics for biologists contains articles on many of the points above.

Software and code

Policy information about availability of computer code

Data collection	(1) EPU (ThermoFischer Scientific; version 2.8.1): software for the authomated cryo-EM data collection. (2) Xcalibur (Thermo Fisher Scientific; version 4.2): to acquire, and interrogate data from the LC-mass spectrometry. (3) BLAST for sequences search.
Data analysis	(1) MotionCor2 (version 2016, before the oficial 1.0.0), Gctf (version 1.06), Gautomatch (version 0.56), SCIPION (version 2.0): for the cryo-EM data preprocessing. (2) RELION (version 3.1), cryoSPARC (version 3.3.1), SPIDER (version 14): for the data processing and post processing. (3) SWISS-MODEL (online server), Coot (version 0.8.9.3) and PHENIX (version 1.20.1), MolProbity (within PHENIX): model building and model refinement. (4) MaxQuant (version 1.5.2.8): proteomics. (5) ClustalX (version 2.0.9) and Clustal Omega (version 1.2.4): sequences alignment. (6) BioEdit (version 7.0.5.3), Mega X (version 10.0.5) and iTOL (version 6.7.2): phylogenetic analysis and visualization. (7) PSIPRED (version 4.0): secondary structure prediction. (8) Chimera (version 1.14) and ChimeraX (version 1.3): map visualization, analysis and figure preparation.

For manuscripts utilizing custom algorithms or software that are central to the research but not yet described in published literature, software must be made available to editors and reviewers. We strongly encourage code deposition in a community repository (e.g. GitHub). See the Nature Portfolio guidelines for submitting code & software for further information.

Data

Policy information about availability of data

All manuscripts must include a data availability statement. This statement should provide the following information, where applicable:

- Accession codes, unique identifiers, or web links for publicly available datasets
- A description of any restrictions on data availability
- For clinical datasets or third party data, please ensure that the statement adheres to our policy

The cryo-EM maps for the 40S, 60S, and 80S with bound tRNAs have been deposited in the Electron Microscopy Data Bank with accession codes EMDB-15674, EMDB-15773, and EMDB-15806, respectively. The atomic models for the 60S, 40S, and actively translating 80S ribosome have been deposited in the Protein Data Bank under accession codes pdb:8auv, pdb:8azw, and pdb:8b2l, respectively. The mass spectrometry proteomics data have been deposited to the ProteomeXchange Consortium via the PRIDE partner repository with the dataset identifier PXD032330.

Dataset 'N. tabacum BX Sierro 2014 BLAST' from the Sol Genomics Network database (https://solgenomics.net) was used to obtain RNA sequences for model building. Starting atomic coordinates used to builds the tobacco 80S ribosome model: pdb:5m1j, pdb:6ek0, pdb:5aj0. Ribosomal models used for comparative analyses during the study: pdb:6y57, pdb:6qnr, pdb:4v88, pdb:7qiz, pdb:4v6w, pdb:4v8p pdb:4bts, pdb:4v9d.
To obtain the sequences the following databases were used: NCBI, Phytozome (https://phytozome.jgi.doe.gov), FernBase (https://www.fernbase.org), ConGenIE (http://congenie.org).

Human research participants

Policy information about studies involving human research participants and Sex and Gender in Research.

Reporting on sex and gender	not applicable
Population characteristics	not applicable
Recruitment	not applicable
Ethics oversight	not applicable

Note that full information on the approval of the study protocol must also be provided in the manuscript.

Field-specific reporting

Please select the one below that is the best fit for your research. If you are not sure, read the appropriate sections before making your selection.

☒ Life sciences ☐ Behavioural & social sciences ☐ Ecological, evolutionary & environmental sciences

For a reference copy of the document with all sections, see nature.com/documents/nr-reporting-summary-flat.pdf

Life sciences study design

All studies must disclose on these points even when the disclosure is negative.

Sample size	The maximum number of usable micrographs (movies recorded: 14,651; as determined by visual inspection and inspection of the power spectra for resolution and good Thon rings) was used, to increase the number of potential particles to the maximum for extensive sorting. Initial particle number was 2,003,888. The maximum number of particles from the full dataset was obtained based on reference-free screening with optimized parameters (particle diameter and threshold).
Data exclusions	Following the 2D classification, 737,462 false-positive particles, representing contaminations, were excluded from the following analysis.
Replication	Due to the nature of the experiment, and typical for the single particle field, no replication of the whole experiment was performed. Individual micrographs shown here are typical for good quality micrographs; during initial inspection, approximately 600 micrographs were inspected.
Randomization	Randomization is not relevant to single-particle cryo-EM.
Blinding	Blinding is not relevant to single-particle cryo-EM.

Reporting for specific materials, systems and methods

We require information from authors about some types of materials, experimental systems and methods used in many studies. Here, indicate whether each material, system or method listed is relevant to your study. If you are not sure if a list item applies to your research, read the appropriate section before selecting a response.

Materials & experimental systems

n/a	Involved in the study
☒	☐ Antibodies
☒	☐ Eukaryotic cell lines
☒	☐ Palaeontology and archaeology
☒	☐ Animals and other organisms
☒	☐ Clinical data
☒	☐ Dual use research of concern

Methods

n/a	Involved in the study
☒	☐ ChIP-seq
☒	☐ Flow cytometry
☒	☐ MRI-based neuroimaging
